# Developmental molecular signatures define *de novo* cortico-brainstem circuit for skilled forelimb movement

**DOI:** 10.21203/rs.3.rs-6150344/v1

**Published:** 2025-03-26

**Authors:** Julia Kaiser, Payal Patel, Sam Fedde, Alexander Lammers, Matthew R Kenwood, Asim Iqbal, Mark Goldberg, Vibhu Sahni

**Affiliations:** 1 Burke Neurological Institute, White Plains, NY, 10605; 2 Tibbling Technologies, Redmond, WA, 98052; 3 Department of Neurology, UT Health Sciences Center San Antonio, San Antonio, TX, USA; 4 Feil Family Brain and Mind Research Institute, Weill Cornell Medicine, New York City, NY, 10065

**Keywords:** development, motor circuitry, sensory circuitry, corticospinal, cortico-brainstem, cortical development, molecular controls over neuronal diversity, skilled movement, axon guidance, subcerebral projection neurons, projection neuron diversity

## Abstract

Skilled movement relies on descending cortical projections to the brainstem and spinal cord. While corticospinal neurons (CSN) have long been recognized for their role in fine motor control, the contribution of cortical projections to the brainstem remains poorly understood. Here, we identify a previously unrecognized direct cortico-brainstem circuit that emerges early in development and persists into adulthood. A subset of subcerebral projection neurons (SCPN) limit their projections to the brainstem from the earliest stages of axon extension without ever extending to the spinal cord. Using FACS purification and single-cell RNA sequencing, we show that these cortico-brainstem neurons (CBN) can be prospectively identified by the expression of Neuropeptide Y (Npy) in development. Functional silencing of Npy+ CBN in adulthood leads to impaired skilled forelimb reaching, demonstrating their essential role in adult motor control. Npy+ CBN project preferentially to rostral brainstem regions, including the midbrain reticular formation. These findings reveal developmental molecular signatures that define cortico-brainstem pathways for adult skilled movement. Our work provides new insights into the developmental logic that establishes descending cortical circuits and opens avenues for targeted investigation of their roles in motor function and recovery after injury.

## Introduction

For skilled movement, neocortical subcerebral projection neurons (SCPN) must make precise connections with subcerebral targets in the brainstem and spinal cord^1–3^. This segmentally specific connectivity enables distinct motor output and functions: cortico-brainstem neurons (CBN), often referred to as “cortico-bulbar” neurons, have long been recognized for their role in controlling face and eye movements by innervating specific brainstem targets, while corticospinal neurons (CSN) are primarily thought to control movement of the limbs, trunk, and legs by innervating their targets in distinct spinal segments^4^. Recent findings have expanded our understanding regarding the role of the brainstem, identifying it as an additional critical center for controlling skilled forelimb movements, including forelimb motor control^5,6^. A substantial amount of input into the brainstem comes from different cortical areas^7,8^. These cortico-brainstem projections can play distinct roles in controlling dexterous movement as compared to corticospinal projections^9^, which themselves vary in control depending on their projection patterns^10,11^. Following central nervous system (CNS) damage, the plasticity of these projections plays a crucial role in motor recovery, suggesting that CBN and CSN contribute differentially to functional motor recovery^12,13^. CBN and CSN reside interdigitated in layer V of cortex and as a result distinguishing these subpopulations has proved to be experimentally challenging. Therefore, how these distinct projections are specified during development, and how they might specifically contribute to distinct functional outputs largely remains unclear.

Early investigations into SCPN axon development suggested that the distinction between CBN and CSN is established via large-scale developmental pruning. During early development, SCPN axons extend more broadly to a larger set of targets than will be retained in the adult. These exuberant projections are then refined through selective axon pruning so that in adulthood, specialized neocortical areas establish projections to specific subsets of targets in the brainstem and spinal cord^14–16^. This pruning to generate area-specific connectivity is controlled by molecular differences between SCPN residing in distinct cortical areas^17^. Using such molecular differences, SCPN can be reprogrammed from spinal-projecting to proximal-projecting types, effectively pruning their axons to target more proximal levels of the neuraxis^18^. Interestingly, these recent investigations also identified a subset of proximal-projecting SCPN that emerge de novo, independent of pruning, suggesting that a subset of SCPN establishes a direct connection to the brainstem, without ever projecting to the spinal cord. An intriguing possibility is that this subset is molecularly specified to be distinct from other SCPN that extend axons to the spinal cord during early development. This would suggest an intrinsic molecular code that underlies SCPN axon extension specificity early in development that limits CBN axons from ever extending to the spinal cord. Such cell-intrinsic molecular mechanisms have been recently identified as controlling SCPN axon extension specificity across the cervical-thoracic transition zone in the spinal cord^19–21^. These results suggest that it might be possible to molecularly distinguish CBN from CSN during initial SCPN axon extension, prior to the large-scale axonal pruning events described above.

In the present study, we establish that there is SCPN axon extension specificity at the brainstem-spinal transition zone from the earliest stages of axon extension. We find that a subset of SCPN limits its axon extension to the brainstem, establishing that there is early differentiation of CBN from CSN. We further establish that CBN can be molecularly differentiated from CSN even within the same cortical areas during early development indicating that axon extension specificity represents a key driver of SCPN molecular diversification. This now provides unprecedented prospective molecular access to these distinct subpopulations throughout development and into maturity. Using this molecular distinction, we selectively silenced CBN (Npy^+^) in adulthood and reveal that this newly identified, developmentally defined cortico-brainstem circuit is essential for skilled forelimb control. Finally, we use this early molecular delineation to identify anatomical differences in brainstem innervation by CBN versus CSN axons, with CBN axons preferentially innervating more rostral brainstem nuclei than CSN axons. These anatomical findings suggest potentially novel circuit level substrates underlying cortical control over skilled forelimb movement. Collectively, our results identify early developmental molecular programs over SCPN diversification, which lays the foundation for participation by distinct SCPN subpopulations in anatomically and functionally distinct circuits at maturity. These findings not only deepen our understanding of SCPN development but also highlight how molecular control of early subpopulation differentiation has lasting implications for adult motor function, providing novel avenues to identify, define, and manipulate previously unknown cortico-brainstem circuits essential for skilled movement control.

## Results

### Cortico-brainstem neurons restrict axon extension to supraspinal levels from the earliest stages of SCPN development.

**Fig. 1:**
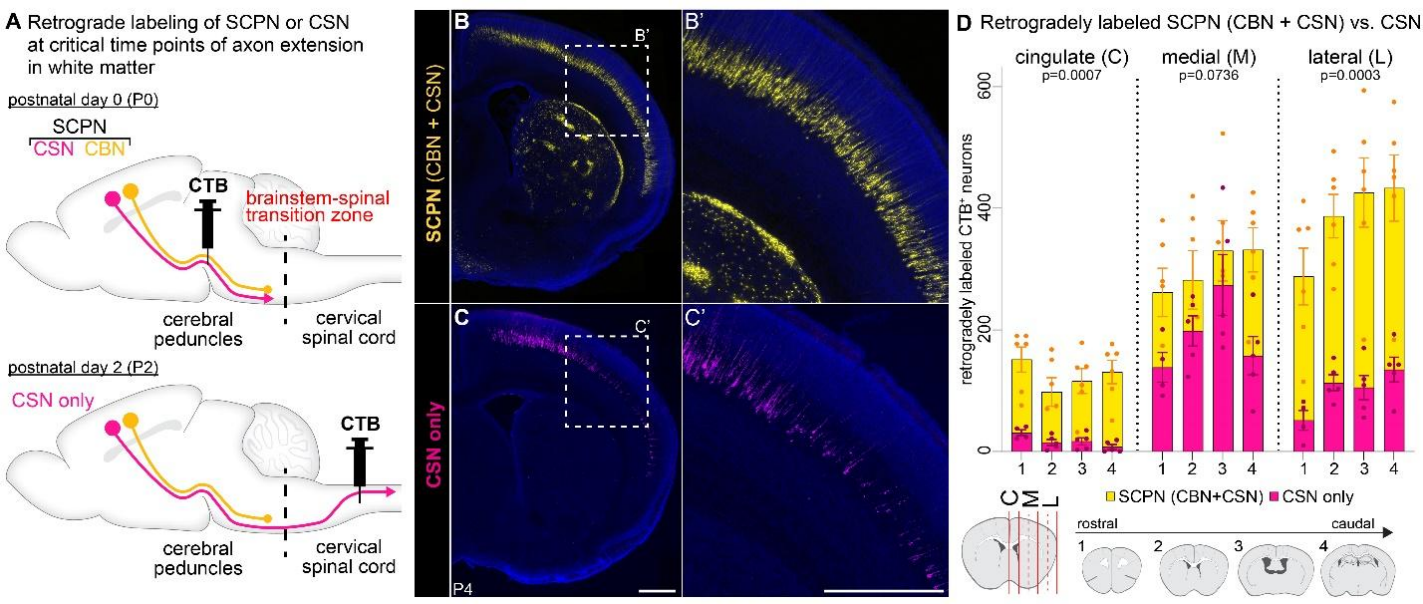
The brainstem-spinal transition zone is established early in development. (A) Retrograde labeling strategy: Subcerebral projection neurons (SCPN) extend axons to subcerebral targets. At P0 (upper panel), SCPN axons can be found at caudal brainstem levels. P0 CTB injection into cerebral peduncles labels all SCPN, including both putative cortico-brainstem neurons (CBN, yellow) that extend only to the brainstem, and corticospinal neurons (CSN, magenta) that extend to the spinal cord. At P2, axons of CSN have extended into the spinal cord; thus, injection of CTB at C2 exclusively labels CSN. (B) Retrogradely labeled SCPN reside throughout the medio-lateral extent of sensorimotor cortex. (C) The majority of labeled CSN reside in medial sensorimotor cortex, with few CSN within the lateral sensorimotor cortex (zoom-in views), (D) Quantification of retrogradely labeled neurons. Almost all SCPN in cingulate cortex are CBN; the majority of CSN reside in medial sensorimotor cortex together with CBN, but only a minority of SCPN in lateral sensorimotor cortex are CSN. SCPN n = 6, CSN n = 5. Statistical difference between SCPN vs. CSN indicated as p-value. Scale bar: 500μm.

Subcerebral projection neurons (SCPN) extend their axons from layer V in neocortex to subcerebral targets along the rostro-caudal axis, eventually innervating either the brainstem (cortico-brainstem projection neurons, CBN) or spinal cord (corticospinal projection neurons, CSN) (Fig. 1A). Recent findings have shown that from the earliest stages of axon extension into the cord, and prior to the formation of any functional connectivity, SCPN exhibit axon extension specificity at the cervical-thoracic transition zone^20^. This transition zone represents a critical region of differential SCPN axon extension, where specific SCPN subpopulations either continue their extension or restrict their axons within defined boundaries. We hypothesized that such axon extension specificity might similarly occur at other transition zones in the white matter across the neuraxis. We therefore analyzed the transition between the brainstem and the cervical spinal cord, i.e., the “brainstem-spinal” transition zone, to investigate whether developing SCPN axons navigating through the brainstem could similarly exhibit distinct axon extension specificity.

We retrogradely labeled SCPN from the white matter at two distinct levels of the neuraxis –the rostral end of the brainstem and the rostral cervical cord– to determine whether a subset of SCPN limits their axon extension to supraspinal levels. We injected cholera toxin B (CTB), a retrograde tracer at specific developmental times to coincide with critical stages of SCPN axon extension: we injected CTB into the tract at the level of the cerebral peduncles at postnatal day 0 (P0), when SCPN axons have reached the brainstem and have not yet reached the spinal cord^22^. This injection therefore labels all SCPN (CBN + CSN) (Fig. 1A, upper panel). Coronal brain sections at P4 find retrogradely labeled SCPN are broadly distributed across layer V spanning the mediolateral extent of the entire sensorimotor cortex (Fig. 1B). In a separate cohort, at P2, when SCPN axons have extended into the cervical spinal cord^22^, we injected CTB into the dorsal funiculus of the cervical spinal cord to selectively label CSN (Fig. 1A, lower panel). Coronal brain sections at P4 find fewer retrogradely labeled CSN from the cervical spinal cord at P2 as compared to retrogradely labeled SCPN from the cerebral peduncles at P0 (Fig. 1C). This indicates that a significant number of SCPN axons extend to the brainstem but do not extend further into the cervical spinal cord (i.e., these are CBN). This finding suggests that at least a subset of CBN restrict axon extension to supraspinal levels from the earliest stages of axon elongation, and that this occurs well before collateral branching or gray matter innervation.

Quantification of retrogradely labeled CBN and CSN across the sensorimotor cortex at P4 revealed that their distribution varies significantly between cortical areas (Fig. 1D). CSN are predominantly located in medial cortex (including primary motor cortex), where they reside intermingled with CBN (SCPN vs. CSN, p=0.0736). In contrast, the cingulate cortex is almost entirely devoid of CSN, with nearly all SCPN in this region being CBN (SCPN vs. CSN, p=0.0007). Similarly, the lateral cortex (including somatosensory cortex) is enriched in CBN, though CSN constituted a minority of the SCPN population in this area (SCPN vs. CSN, p=0.0003). Notably, SCPN in the lateral cortex were evenly distributed throughout the rostro-caudal axis (Fig. 1D). Collectively, these results demonstrate that CBN axons limit their axon extension to supraspinal levels from the earliest stages of development, establishing the brainstem-spinal transition zone as a critical site for differential SCPN axon extension specificity.

### Molecular specification of SCPN subpopulations occurs across two independent axes: axon extension specificity and cortical location.

**Extended Data Fig. 1:**
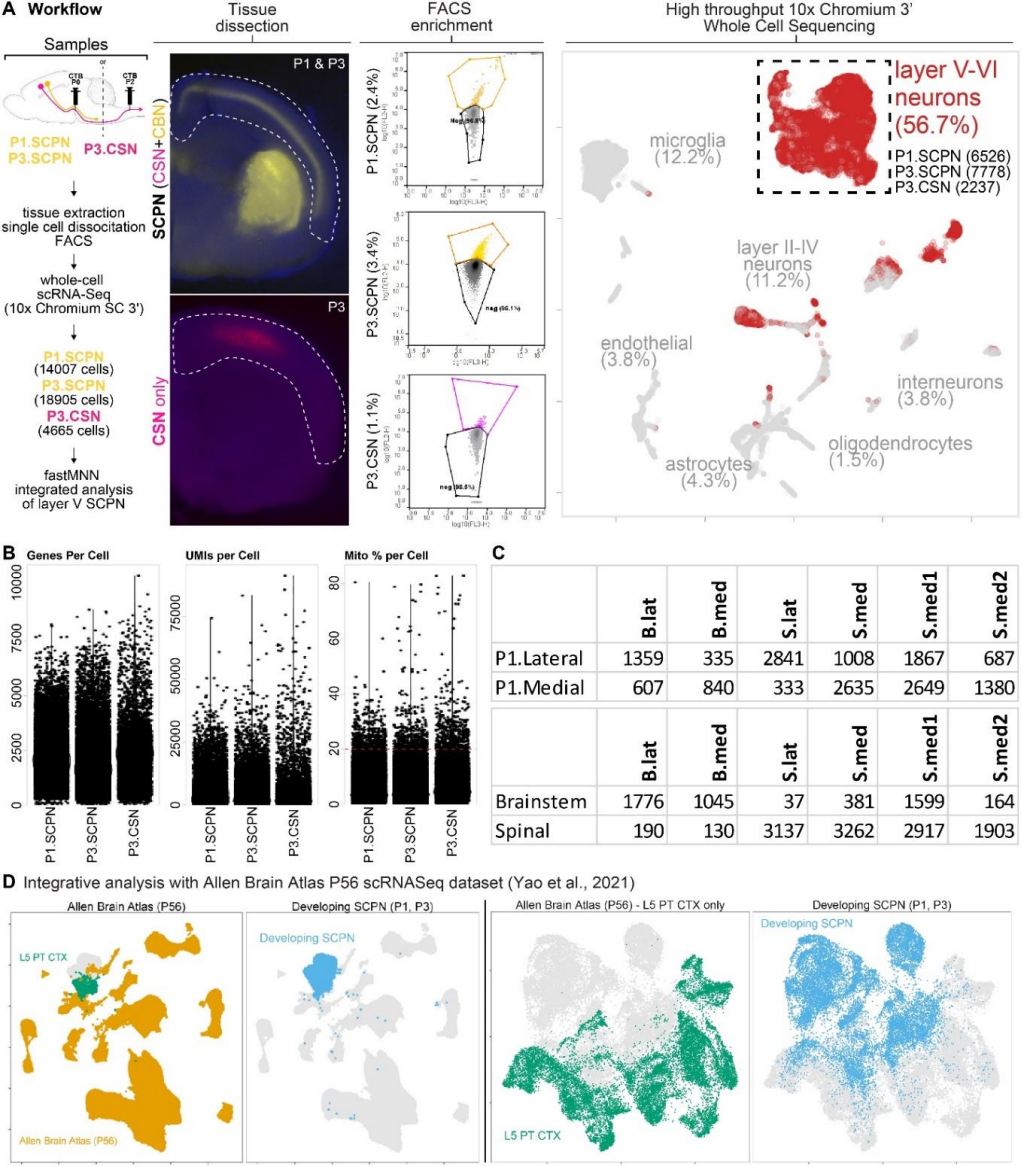
Enrichment of retrogradely labeled SCPN for whole-cell scRNAseq at critical developmental times of axon extension specificity. (A) Workflow for FACS purification and scRNA-seq pipeline of SCPN vs. CSN, demonstrating effective enrichment of whole cell SCPN across groups (P1.SCPN, P3.SCPN, P3.CSN). (B) Quality control metrics, including genes per cell, UMIs per cell, and mitochondrial percentage per cell. Note that these metrics remain consistent across all groups. (C) Cell distribution by cluster, classified into lateral/medial (top) and brainstem/spinal (bottom) categories. (D) Integration with the adult Allen Brain Atlas (P56) reveals developmental SCPN share characteristics with Layer 5 pyramidal neurons (L5 PT CTX) (left two panels), yet exhibit distinct developmental profiles (right two panels): Out of 16541 developing SCPN (11%), 1886 overlapped with L5 PT CTX, and vice versa out of 17260 L5 PT CTX neurons, only 557 overlapped with developing SCPN (3%).

**Fig. 2:**
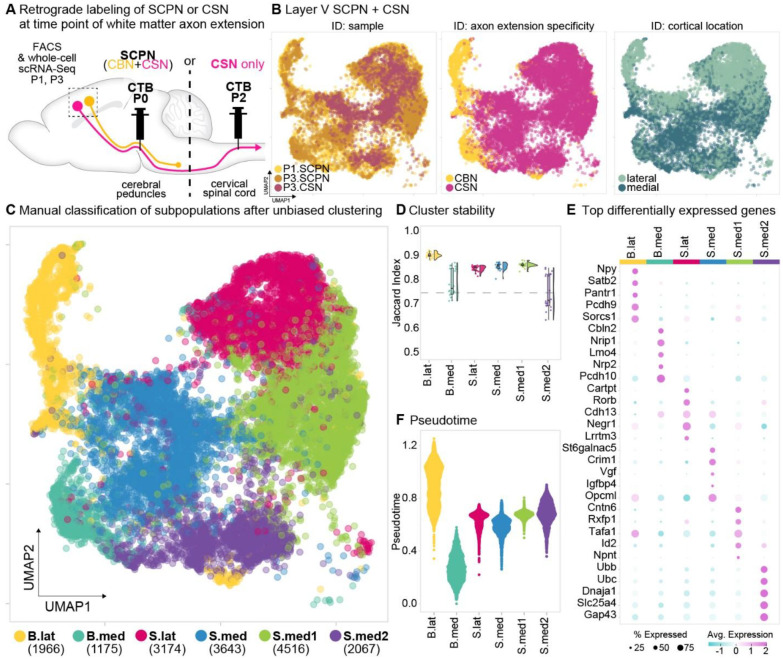
Molecular specification of SCPN subpopulations based on axon extension specificity and cortical location. (A) Experimental scheme: SCPN or CSN were retrogradely labeled (SCPN at P0, CSN at P2), cortices were dissociated at P1 (SCPN) or P3 (SCPN or CSN) and whole cell suspensions were FACS purified for single cell sequencing. 3 samples were collected: P1.SCPN, P3.SCPN, P3.CSN. (B) Layer V SCPN/CSN pseudocolor coded based on sample (left panel), axon extension specificity (middle panel) or cortical location (right panel). (C) Manual annotation of 6 unbiased clusters (kmeans50) based on location in medial (”MED”) vs. lateral (”LAT”) cortex and axon extension specificity (clusters with cells only from SCPN samples were classified as “CBN” (brainstem-projecting, ”B”), while clusters with cells from both SCPN and CSN samples were classified as “CSN” (spinal-projecting, ”S”)). (D) cluster stability highlights B.LAT as the most stable cluster (E) Heatmap of top 5 differentially expressed genes between the 6 clusters. Npy and Cartpt are the top differentially expressed genes for B.LAT and S.LAT, respectively. Top 250 genes for each cluster can be found in Supplementary Table 1. (F) Pseudotime analysis suggests that B.LAT neurons are most differentiated in comparison with all other SCPN clusters.

The striking axon extension specificity between CBN and CSN suggests that these SCPN subpopulations are molecularly specified early in development to exhibit differential axon extension. To investigate the underlying molecular signatures that distinguish developing CBN from CSN, we retrogradely labeled either all SCPN from the cerebral peduncles (CBN + CSN), or CSN only (from the cervical cord) (Fig. 2A, Extended Data Fig. 1A). We injected CTB at the same time points as described above– either into the cerebral peduncles at P0 or into the cervical cord at P2– and FACS-purified retrogradely labeled SCPN or CSN one day later for whole cell single-cell RNA-sequencing (scRNA-Seq). We next integrated these datasets of critical developmental times (SCPN at P1, P3, and CSN at P3) (Fig. 2B, ID: Sample). Our hypothesis was that in this integrated molecular space we would identify CSN as a subset of the overall population of SCPN, which also includes CBN. We further reasoned that this approach would enable identification of molecular distinctions between CBN and CSN. Our goal, therefore, was to identify molecular differences that encapsulate these critical developmental stages of white matter axon extension: P1, just as CSN axons are beginning to reach the spinal cord, while CBN axons have limited extension to the brainstem, and P3, shortly after CSN extend axons into the spinal cord^22^. We therefore analyzed gene expression profiles in the integrated molecular space to find molecular correlates of the differences in axon extension specificity between CBN and CSN.

The scRNA-Seq analysis yielded a median of 3,498 unique molecular identifiers (UMIs) per cell, with a median of 1,904 detected genes and a median of 6.98% mitochondrial gene content (Extended Data Fig. 1B). Using an available scRNA-Seq dataset from the P0 mouse cortex^23^, we confirmed that our FACS-enriched population predominantly consisted of layer V neurons (Extended Data Fig. 1A, dashed box), which were used for downstream analysis. To confirm SCPN enrichment, we also compared these neurons with the publicly available adult Allen Brain Atlas (P56 Whole Cortex & hippocampus)^24^. We built an integrated dimensional space combining our single cell dataset of FACS-purified SCPN in development with all identified cell types in adult cortex. As expected, we find that the FACS-purified developing SCPN in our dataset are most closely related to layer 5 PT glutamatergic neurons in the adult cortex, further confirming SCPN enrichment (Extended Data Fig. 1D, left panel).

We next performed unbiased clustering of these neurons and identified six transcriptionally distinct and stable clusters (Fig. 2D, E). We hypothesized that the diversity in axonal extension might, at least partially, contribute to these transcriptional differences. We previously identified that SCPN in medial versus lateral cortex exhibit axonal projections to distinct segmental levels. SCPN in lateral cortex extend axons exclusively to the brainstem and cervical spinal cord (bulbar-cervical), whereas SCPN in medial cortex are relatively more heterogeneous, and distinct subsets extend axons to either bulbar-cervical or thoraco-lumbar segments^20^. We therefore investigated whether the six molecularly distinct SCPN clusters observed in our scRNA-Seq dataset correlate with differences in SCPN cortical location. We used transcriptional profiling data from our prior study, which identified differentially expressed genes between medial vs. lateral SCPN^20^, to annotate each cluster based on whether SCPN in that cluster reside in medial versus lateral cortex (Fig. 2B, ID: cortical location, Extended Data Fig. 1C). We also classified each of the six clusters based on axon extension specificity. For this, we annotated clusters by sample subtype: clusters that contain cells exclusively in SCPN, but not CSN samples, were annotated as “CBN”, while clusters present in both SCPN and CSN samples were annotated as “CSN” (Fig. 2B, ID: axon extension specificity, Extended Data Fig. 1C). Based on these annotations, we manually classified the clusters by their axon extension specificity (cortico-brainstem “B” vs. corticospinal “S”) and cortical location (medial “med” vs. lateral “lat”) (Fig. 2C). Interestingly, cortical location and axon extension specificity of SCPN clusters segregate along orthogonal axes (Fig. 2B): Medial and lateral SCPN clusters are distinguished along an axis distinct from the one separating CBN from CSN. This indicates that CBN and CSN can be molecularly distinguished within both medial and lateral cortex, suggesting parallel but distinct molecular programs encode SCPN cortical location and axon targeting specificity.

Consistent with previous findings^20^, we find that more transcriptionally distinct SCPN clusters arise from medial cortex (4 out of 6 total) compared to those in lateral cortex (2 out of 6). This likely reflects the greater diversity of subcerebral projection targets within medial SCPN. Specifically, medial SCPN were divided into four distinct subclusters: three corticospinal clusters (S.med, S.med1, and S.med2) and one cortico-brainstem cluster (B.med). In contrast, lateral SCPN segregated into two clusters—one cortico-brainstem (B.lat) and one corticospinal (S.lat) (purple and yellow clusters, Fig. 2C)— further supporting our previous findings that subcerebral projections from lateral cortex are relatively more homogeneous than those arising from medial cortex. This is further supported by the cluster stability assessment (Fig. 2D), which shows that the two lateral SCPN clusters exhibit greater overall stability than the medial SCPN clusters. Given that lateral SCPN axons extend exclusively to more rostral levels of the neuraxis (brainstem and cervical cord)^20^, the spinal lateral cluster likely represents cervical-projecting CSN (CSN_C-lat_).

We next investigated whether all SCPN are equally differentiated at this early developmental time. We hypothesized that, since CBN limit axon extension at a time when CSN are still extending axons, CBN might be more differentiated than CSN. We used pseudotime analysis to examine the developmental trajectories of these distinct SCPN clusters and compare their differentiation status relative to each other (Fig. 2F). Notably, the brainstem lateral cluster emerged as the most differentiated among all SCPN subpopulations, with no significant differentiation differences across the remaining clusters. This finding suggests that, although not all CBN are more differentiated than CSN, CBN in lateral cortex are specifically more developmentally advanced than all other SCPN at this early developmental stage.

To investigate the unique developmental molecular signature of SCPN subpopulations, we identified the top differentially expressed genes across all clusters (Fig. 2E, Supplementary Table 1). Notably, we find *Crim1* and *St6galnac5*, two genes previously associated with CSN in medial cortex^19,20^, in the cluster manually annotated as “S.med1” (Fig. 2F). In lateral cortex, *Neuropeptide Y* (*Npy*) and *CART prepropeptide* (*Cartpt*), were the top genes differentially expressed by the two distinct clusters “B.lat” and “S.lat”, respectively (Fig. 2E).

### Npy and Cartpt expression defines molecularly distinct SCPN subpopulations in postnatal cortex

**Fig. 3:**
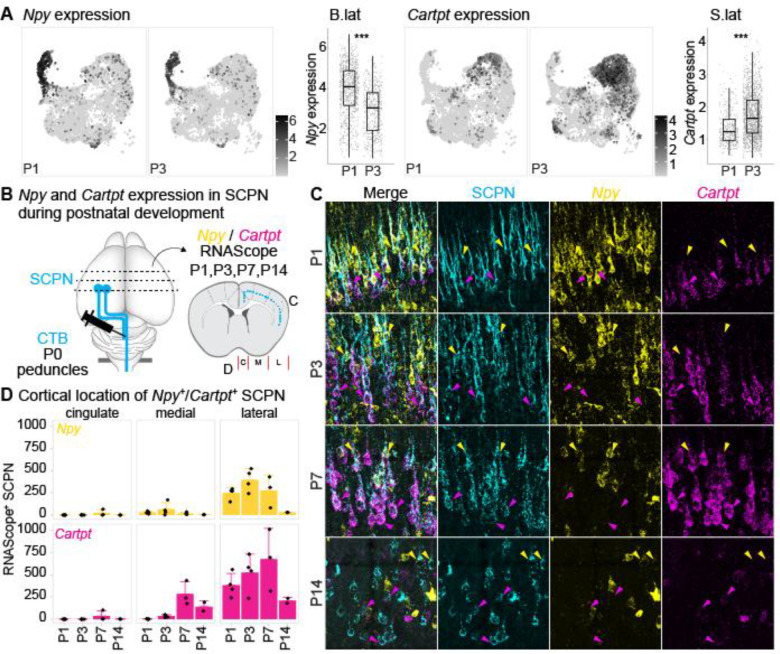
Temporal and spatial expression profiles of Npy and Cartpt delineate distinct SCPN subpopulations during postnatal development. (A) UMAP visualization shows that Npy is specifically enriched in the B.LAT cluster, while Cartpt is enriched in the S.LAT cluster. (B) Experimental design: SCPN were retrogradely labeled at P0, and Npy and Cartpt expression in cortex was analyzed using RNAscope at P1, P3, P7 and P14. (C) Representative images of RNAscope: Npy (yellow) and Cartpt (magenta) expression in SCPN (cyan). (D) Quantification of Npy^+^ and Cartpt^+^ SCPN across cingulate, medial, and lateral cortex. Npy^+^ SCPN are predominantly localized in lateral cortex, with an increase in expression from P1 to P3 and a subsequent decrease. Cartpt is also enriched in lateral cortex with a peak at P7, with some expression in medial cortex. *P < 0.05, **P < 0.01, ***P < 0.001.

To validate and confirm the annotation of the distinct SCPN clusters by cortical location and axon extension specificity, we focused on SCPN clusters in lateral cortex. ScRNA-Seq analysis identified *Npy* and *Cartpt* as the top differentially expressed genes. *Npy* expression is predominantly localized in the “B.lat” cluster with an enrichment at P1 (mean expression level 3.93 ± 1.25 at P1 vs. 2.92 ± 1.14 at P3), while *Cartpt* expression was enriched in the “S.lat” cluster predominantly at P3 (Fig. 3A, mean expression level 1.37 ± 0.52 at P1 vs. 1.74 ± 0.69 at P3). We performed RNAScope in-situ hybridization to assess the temporal and spatial expression profiles of *Npy* and *Cartpt* at selected postnatal time points (P1, P3, P7, P14) (Fig. 3B). While *Npy* is typically known to be expressed by cortical interneurons^25^, recent studies have identified *Npy* expression in a subset of excitatory projection neurons during early cortical development^26^. To specifically investigate *Npy* and *Cartpt* expression by SCPN, we combined RNAScope with retrograde labeling from the cerebral peduncles (see Fig. 1A) and quantified the number of *Npy*^+^ vs. *Cartpt*^+^ SCPN.

**Extended Data Fig. 2.**
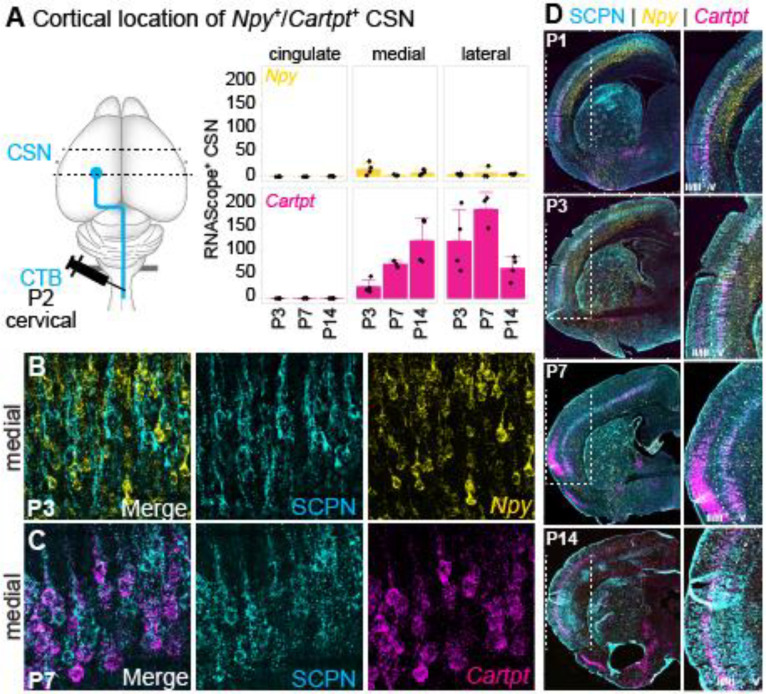
Genes expressed by distinct SCPN populations in the same cortical location exhibit different temporal dynamics of expression. (A) Retrogradely labeled CSN (from cervical C2). Quantification of numbers of labeled CSN with RNAScope signal for Npy and Cartpt show minimal Npy^+^ CSN, while the numbers of Cartpt^+^ CSN follows a similar trend as Cartpt^+^ SCPN, predominantly located in lateral cortex. (B) Representative image of the medial cortex at P3 (peak Npy expression) shows no overlap between SCPN (CTB^+^) and Npy RNAScope signal. (C) Representative image of the medial cortex at P7 (peak Cartpt expression) shows Cartpt RNAScope signal overlapping with few CTB^+^ SCPN. (D) Overview of CTB^+^ SCPN, Npy, and Cartpt distribution across medial-lateral axis at P1, P3, P7, and P14.

Overall, while *Npy* expression remained high in cortex across the different time points, the number of *Npy*^+^ SCPN in lateral cortex increased from P1 to P3, followed by a decline from P3 to P14 (Fig. 3C, yellow channel, Fig. 3D, upper panel). Similarly, the number of *Cartpt*^+^ SCPN in lateral cortex increased from P1 to P7 and decreased thereafter (Fig. 3C, magenta channel; Fig. 3D, lower panel). Notably, *Npy* and *Cartpt* expression is largely non-overlapping, indicating that these genes are expressed by distinct SCPN populations in lateral cortex (Fig. 3C). We quantified the numbers of *Npy*^+^ and *Cartpt*^+^ SCPN in developing neocortex: *Npy*^+^ SCPN are predominantly located in lateral cortex and almost no Npy^+^ SCPN are located in cingulate or medial cortex (Fig. 3D, upper panel, Extended Data Fig. 2B,D). *Cartpt*^+^ SCPN can be found in medial cortex at P7; however the overall majority reside in lateral cortex (Fig. 3D, lower panel, Extended Data Fig. 2C,D). These expression analyses therefore validate our annotation of “B.lat” and “S.lat” clusters in the scRNAseq dataset as residing in lateral cortex.

We next sought to validate our annotation of axon extension by determining whether, at this early developmental stage, *Npy*^+^ SCPN correspond to CBN, while *Cartpt*^+^ SCPN project to the spinal cord as CSN. To test this, we performed retrograde labeling from the cervical spinal cord to identify CSN, combined with RNAscope analysis, to quantify *Npy*^+^ vs. *Cartpt*^+^ CSN. Consistent with our annotations, we find almost no *Npy* expression in retrogradely labeled CSN (Extended Data Fig. 2A). In striking contrast, *Cartpt* is expressed in CSN, and this expression follows a similar temporal and spatial profile as observed in SCPN (Extended Data Fig. 2A–C). Therefore, *Npy* and *Cartpt* are specifically expressed in distinct SCPN subpopulations during early postnatal development. While both subpopulations are in lateral cortex, they exhibit striking targeting differences at this initial stage of axon extension.

**Extended Data Fig. 3:**
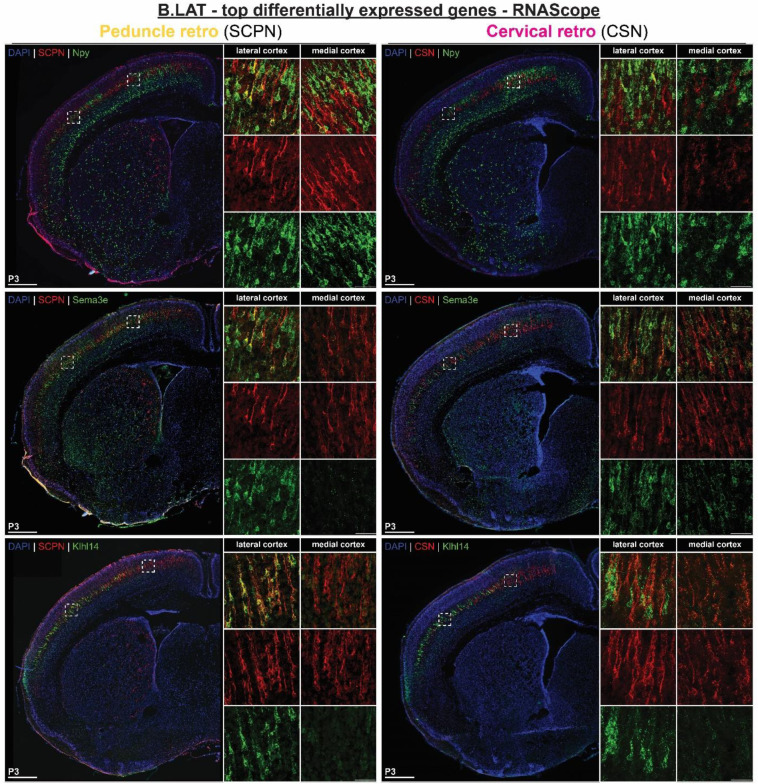
CBN-specific gene expression in developing lateral cortex. Npy and Sema3e are predominantly expressed in CBN with minimal expression in CSN, while Klhl14 is expressed by both CBN and CSN in lateral cortex. Coronal sections of P3 mouse brains retrogradely labeled from either the cerebral peduncle (Peduncle retro) or cervical spinal cord (Cervical retro) combined with RNAscope in situ hybridization for Npy (top row), and Sema3e (middle row) and Klhl14 (bottom row). Npy and Sema3e demonstrate co-localization with retrogradely labeled SCPN (left) but not CSN (right), confirming that these genes are largely expressed in CBN. Notably, Klhl14 shows expression by SCPN and some overlap with CSN.

**Extended Data Fig. 4:**
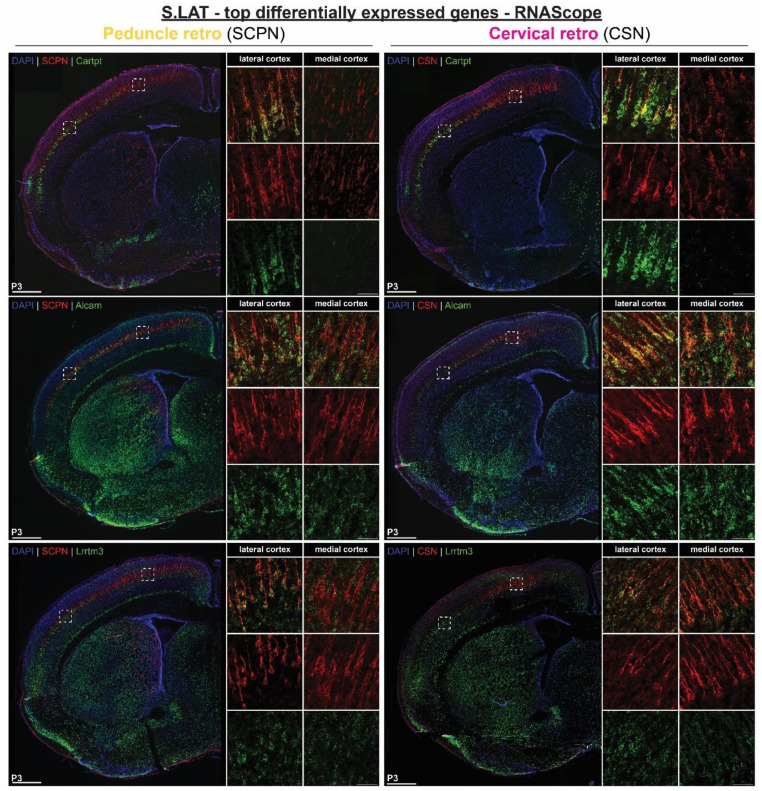
Cartpt, Alcam, and Lrrtm3 are expressed by CSN in lateral cortex. Coronal sections of P3 mouse brains retrogradely labeled from either the cerebral peduncle (Peduncle retro) or cervical spinal cord (Cervical retro) combined with RNAScope in situ hybridization for Cartpt (top row), Alcam (middle row), and Lrrtm3 (bottom row). All three genes are co-localized with both retrogradely labeled SCPN (left) and CSN (right), confirming specificity of expression by CSN in lateral cortex.

Expression analysis of other top differentially expressed genes in lateral cortex also validate our scRNAseq annotations. *Klhl14*, previously identified as expressed by SCPN in lateral cortex^19,20^, is expressed by both CBN and CSNc (Extended Data Fig. 3). This indicates that although *Klhl14* is expressed by the broader population of SCPN in lateral cortex, it does not distinguish between CBN and CSNc, as we recently validated using a new Klhl14-Cre knock in mouse line^27^. In contrast, other newly identified genes unique to either “S.lat” or “B.lat” clusters are only expressed in lateral cortex and indeed distinguish these distinct SCPN subpopulations. “B.lat” gene *Sema3e*, similar to Npy, is expressed only by retrogradely labeled SCPN and not CSN, indicating specific expression by CBN (Extended Data Fig. 3), while “S.lat” genes (*Alcam* and *Lrrtm3*) are expressed by both retrogradely SCPN and CSN in lateral cortex (Extended Data Fig. 4). Collectively, these results identify novel genes that delineate developing CSN_C-lat_ from CBN_lat_. Taken together, these results confirm the annotation of cortical location and axon extension specificity delineating “B.lat” and “S.lat” clusters in our dataset. To our knowledge, these genes provide the first known molecular identifiers that distinguish cervical-projecting CSN from CBN at these earliest stages of SCPN axon extension into the spinal cord.

### Npy^+^ and Cartpt^+^ SCPN are distinct subpopulations in lateral cortex

The RNAscope analyses identified that Npy and Cartpt delineate axon extension differences between CBN and CSNc at the brainstem-spinal transition zone early in development. Our analyses also established that *Npy* and *Cartpt* expression is highest at P3 and declines thereafter, suggesting that this molecular distinction during early development may not persist into adulthood. Consistent with this, we find that when we compared our dataset of developing SCPN with only the L5 PT CTX cluster from the Allen Brain Atlas^24^, there is minimal overlap (~7% of total cells). Together, these results indicate that early molecular programs delinate axon extension during development, but their expression declines during development. This raises two distinct possibilities, each with vastly different implications for how early axon extension specificity at the brainstem-spinal transition zone shapes adult circuitry. One possibility is that initial differences in axon extension between CBN and CSN are transient and do not persist into adulthood. In this case, Npy^+^ CBN might retain the potential to extend spinal projections later, becoming CSN in adulthood. If so, the molecular cues governing early axon extension specificity would no longer be present in adulthood, and a distinct set of molecules would then subsequently control the specificity of CBN versus CSN axon projections. Alternatively, early axon extension specificity may establish long-term projection patterns with molecular programs initiating permanent circuit distinctions even as their expression diminishes over time. In this case, Npy^+^ CBN would maintain their exclusive projections to the brainstem in maturity and never extend axons to the spinal cord.

**Fig. 4:**
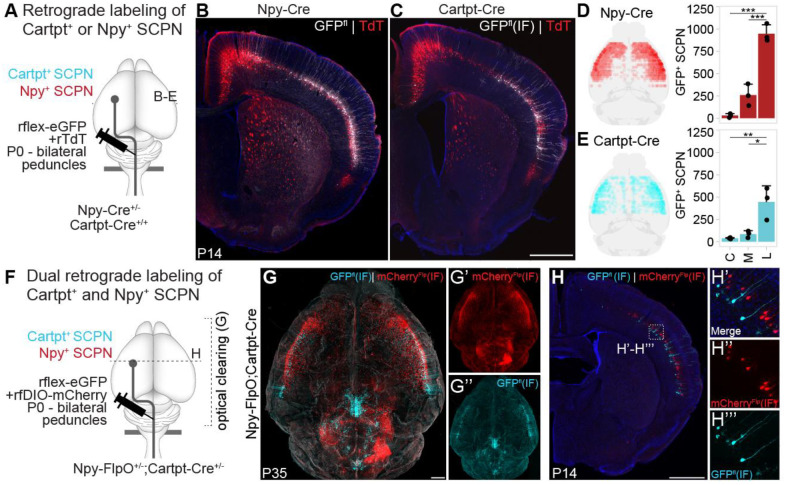
Npy^+^ and Cartpt^+^ SCPN are distinct subpopulations in lateral cortex. (A) Experimental design: Cre-dependent retro-AAVs (GFP) were co-injected with control retro-AAV (TdTomato, TdT) at P0 into cerebral peduncles to retrogradely label Cartpt^+^ or Npy^+^ SCPN in Npy-IRES-Cre or Cartpt-IRES-Cre mice, respectively. (B,C) Coronal section of P14 brains showing broad distribution of control AAV (TdT+) labeling of all SCPN, with Cre-dependent GFP expression specifically in lateral cortex of Npy-Cre (B) and Cartpt-Cre mice (C). (D,E) Quantification of GFP+ SCPN in Npy-Cre (D) or Cartpt-Cre mice (E) shows enrichment of both Npy^+^ and Cartpt^+^ SCPN in lateral cortex. (F) Experimental design for dual retrograde labeling of Cartpt^+^ and Npy^+^ SCPN using Npy-FlpO;Cartpt-Cre double transgenic mice. Cre- and FlpO-dependent retro-AAVs were co-injected into cerebral peduncles at P0. (G) Whole-brain optical clearing and imaging of dual-labeled Npy-FlpO;Cartpt-Cre mice at P35, showing Cartpt^+^ (GFP) and Npy^+^ (mCherry) SCPN enriched in lateral cortex. (H) Coronal section of P14 Npy-FlpO;Cartpt-Cre mouse brain showing Cartpt^+^ (GFP) and Npy^+^ (mCherry) SCPN are distinct subpopulations. *P < 0.05, **P < 0.01, ***P < 0.001.

To distinguish between these possibilities, we used knock-in Cre lines to determine whether this early molecular delineation between developing Npy^+^ vs. Cartpt^+^ SCPN results in persistent and durable differences in axon extension. For this, we first sought to establish whether the Cre mouse lines recapitulated the findings from our RNAscope expression analyses. We therefore performed conditional retrograde labeling in either Npy-Cre or Cartpt-Cre knock-in mouse lines at P0 to confirm whether Cre+ SCPN in these individual mouse lines reside in lateral cortex (Fig. 4A). As expected, these results confirmed that the majority of Npy^+^ and Cartpt^+^ SCPN in the respective Cre mouse lines reside in lateral cortex at P14. In Npy-Cre mice, we find a majority of labeled SCPN are in lateral cortex, with no Npy^+^ SCPN in cingulate, and only a minority in medial cortex (predominantly at rostral levels) (Fig. 4B, D - cell numbers: cingulate 30 ± 22, medial 250.3 ± 122.2, lateral 947.7 ± 100.4). In Cartpt-Cre mice, Cartpt^+^ SCPN are similarly predominantly in lateral cortex (Fig. 4C, E - cell numbers: cingulate 38.3 ± 7.5, medial 85.3 ± 38.1, lateral 444.3 ± 182.5). These results validate that Cre expression in these individual mouse lines faithfully recapitulates endogenous expression of both marker genes.

Since these experiments were performed using different mouse lines, they could not confirm whether *Npy*^+^ and *Cartpt*^+^ SCPN represent distinct subpopulations. Thus, we crossed Npy-FlpO and Cartpt-Cre mice to generate Npy-FlpO;Cartpt-Cre double transgenic mice and then performed dual conditional retrograde labeling. We co-injected both FlpO- and Cre-dependent retro-AAVs at P0 from the cerebral peduncles in these double transgenic mice to simultaneously visualize Npy^+^ (mCherry) and Cartpt^+^ (eGFP) SCPN in the cortex of the same mouse (Fig. 4F). Optical clearing at P35 confirms that both Npy^+^ and Cartpt^+^ SCPN are located in lateral cortex (Fig. 4G–G”). Importantly, we find no overlap between Npy^+^ and Cartpt^+^ SCPN, confirming that these are indeed distinct SCPN subpopulations in lateral cortex that are molecularly distinguishable at this early developmental time (Fig. 4H–H”). These results indicate that this molecular delineation extends beyond areal distinctions, since both subpopulations share the same cortical location.

### Striking axon extension specificity delineates Npy^+^ SCPN as CBN, and Cartpt^+^ SCPN as CSN_c_

**Extended Data Fig. 5:**
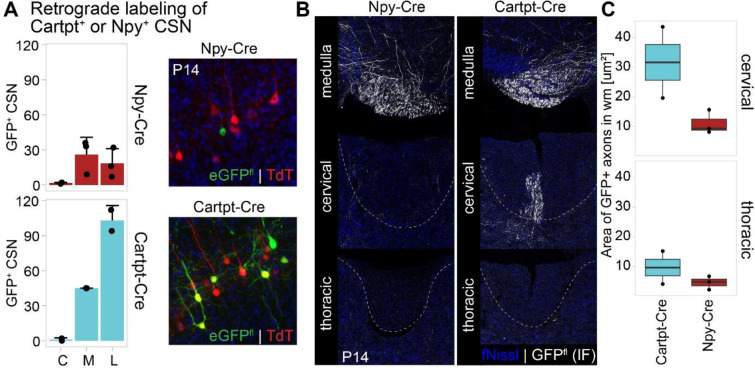
Developmental axon extension specificity between Npy^+^ vs. Cartpt^+^ SCPN persists into maturity. (A) Retrograde labeling from the spinal cord in Npy-Cre and Cartpt-Cre mice reveals few Npy^+^ CSN at P14, in contrast to Cartpt^+^ CSN. Cartpt^+^ CSN are predominantly in lateral cortex. Representative images show positively labeled neurons. (B) Axial sections of anterogradely labeled Npy^+^ CBN (Npy-Cre) and Cartpt^+^ CSN (Cartpt-Cre) at the medulla, cervical, and thoracic spinal cord levels (white matter tract depicted, with labeled axons). Quantification indicates a sharp decline in Npy^+^ axons from the medulla to the cervical cord, while Cartpt^+^ axons are evident in the cervical cord but absent in the thoracic cord.

**Fig. 5:**
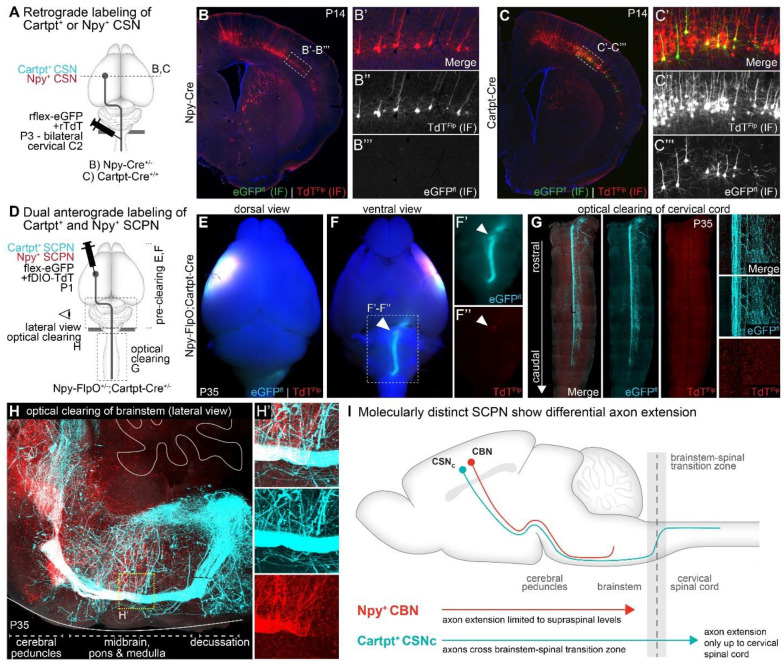
Npy^+^ and Cartpt^+^ SCPN in lateral cortex exhibit striking and persistent axon extension specificity. (A) Schematic of experimental strategy of retrograde labeling: At P3, conditional Cre-dependent (flex-GFP) alongside control (TdT) retro-AAVs were injected into the cervical spinal cord (C2) in Npy-Cre or Cartpt-Cre mice. (B) Coronal section of an injected Npy-Cre mouse brain at P14 shows only control TdT-labeled SCPN, but no GFP labeling, indicating that Npy^+^ SCPN do not project their axons beyond the brainstem. (C) Coronal section of an injected Cartpt-Cre mouse brain at P14 shows robust retrograde labeling of Cartpt^+^ CSN (flex-GFP) from the cervical spinal cord. (D) Schematic of anterograde labeling at P1 by injecting two conditional AAVs (FlpO-dependent TdT, Cre-dependent GFP) into lateral cortex of Npy-FlpO;Cartpt-Cre double transgenic mice. (E-F) Dorsal and ventral whole mount views of an injected brain from a Npy-FlpO;Cartpt-Cre mouse at P14, showing the distinct trajectories of Cartpt^+^ SCPN axons (GFP, cyan) extending to the spinal cord and Npy^+^ SCPN axons (TdT, red) terminate within brainstem. (G-G”) Cervical spinal cord after optical clearing from a P35 Npy-FlpO;Cartpt-Cre mouse. Cartpt^+^ CSN axons (GFP, cyan) extend into the spinal cord, while Npy^+^ SCPN axons (TdT, red) are not present (white arrow). (H) Lateral view of an optically cleared brainstem from a P35 Npy-FlpO;Cartpt-Cre mouse showing the termination of Npy^+^ SCPN axons in the brainstem. Cartpt^+^ SCPN axons continue beyond the brainstem, past the pyramidal decussation and into the spinal cord. High magnification images (H’) highlight the distinct trajectories of these axons. See also Supplementary Video 1. (I) Proposed model of developmental control over SCPN axon extension specificity in lateral cortex: Npy^+^ CBN limit their axon extension to the brainstem, while Cartpt^+^ CSN_c_ extend axons past the brainstem into the cervical spinal cord.

RNAscope *in situ* hybridization analyses had established that, during early postnatal development, Cartpt^+^ SCPN extend axons to the spinal cord while Npy^+^ SCPN do not. We next investigated whether we observed similar axon extension differences between the two subpopulations using the respective Cre mouse lines. For this, we performed retrograde labeling from the cervical spinal cord (C2) in Npy-Cre and Cartpt-Cre mice at P3, followed by analysis at P14 (Fig. 5A). In Cartpt-Cre mice, we find robust retrograde labeling of eGFP^+^ CSN in lateral cortex, indicating that Cartpt-Cre+ SCPN extend axons into the spinal cord (Fig. 5C). In contrast, we find very few eGFP^+^ CSN in Npy-Cre mice, reinforcing the findings from the RNAscope analyses that *Npy*^+^ SCPN do not extend axons beyond the brainstem at this early developmental time (Fig. 5C, Extended Data Fig. 5A). These findings indicate that Cartpt^+^ SCPN project to the spinal cord, while Npy^+^ SCPN limit their axon extension to supraspinal levels. These results validate our expression analyses and establish that Cre expression in these individual Cre mouse lines faithfully recapitulates the anatomical differences between Npy^+^ vs. Cartpt^+^ SCPN, both in their cortical location and their early developmental differences in axon extension specificity.

Having validated this early developmental delineation via these Cre mouse lines, we could now directly address the two distinct possibilities outlined above regarding the contribution of early axon extension specificity to adult circuitry. We next investigated whether this early difference in axon extension specificity between these two molecularly distinct SCPN subpopulations is maintained into maturity. We performed conditional anterograde labeling of Npy^+^ vs. Cartpt^+^ SCPN in Npy-Cre or Cartpt-Cre mice and then quantified axon extension in axial sections at the level of the medulla, cervical cord and thoracic cord at P14 (Extended Data Fig. 5B,C). We find that Npy^+^ SCPN axons are predominantly restricted to medulla, with very few axons detected in the dorsal funiculus at cervical or thoracic segments. In contrast, Cartpt^+^ SCPN extend axons past the brainstem into the cervical, but not thoracic cord. This indicates that the early differences in axon extension specificity between these two subpopulations are maintained into maturity.

To comprehensively investigate the trajectory of axon extension of Npy^+^ vs. Cartpt^+^ SCPN at maturity, we next performed conditional anterograde labeling in Npy-FlpO;Cartpt-Cre double transgenic mice. We co-injected FlpO- and Cre-dependent AAVs into the lateral cortex of these mice at P1 and analyzed the axonal projections at P35 (Fig. 5D). This allowed us to directly compare axon trajectories of these distinct SCPN subpopulations residing in the same area of lateral cortex (Fig. 5F). A ventral view of the wholemount brainstem from these injected mice confirms that Cartpt^+^ SCPN axons extend through the brainstem reaching its caudal most levels where they cross to the dorsal side toward the cervical spinal cord (Fig. 5F+F’, white arrow). In contrast, Npy^+^ SCPN axons terminate within the brainstem, with no evidence of extension to the spinal cord (Fig. 5F+F”, white arrow). We also used optical clearing to highlight this striking difference in axon extension (Fig. 5H, H’, Supplementary Video 1): Npy^+^ SCPN axons terminate extension within the brainstem, and do not extend into the spinal cord, while Cartpt^+^ SCPN axons continue through this transition zone and extend into the spinal cord. We also examined the cervical cord from the double transgenic mouse using optical clearing and find 1) no Npy^+^ SCPN axons, and 2) that Cartpt^+^ SCPN axons limit their extension to the cervical spinal cord (Fig. 5G). Collectively, these findings emphasize the striking axon extension specificity exhibited by these distinct SCPN subpopulations that both reside in lateral cortex: Npy^+^ SCPN limit their axon extension to the brainstem, i.e. Npy^+^ SCPN are CBN and Cartpt^+^ SCPN axons extend past the brainstem to the cervical spinal cord and not beyond, i.e. Cartpt^+^ SCPN are CSNc.

Our findings propose a new model for the developmental regulation of SCPN axon extension, wherein not all developing SCPN axons extend to the spinal cord. Instead, specific SCPN subpopulations consistently and durably restrict their axonal projections to supraspinal levels throughout development and into maturity. These subpopulations are not determined stochastically but are instead molecularly defined during development (Fig. 5I): Npy^+^ CBN are molecularly specified to restrict their axon targeting to supraspinal levels from the earliest stages of developmental axon extension. In contrast, Cartpt^+^ CSN_c_ extend axons beyond the brainstem, projecting to the cervical spinal cord. This further indicates that there is molecular delineation underlying SCPN axon extension that is not mediated via areal regulation alone. Even though both Npy^+^ CBN and Cartpt^+^ CSNc reside in lateral cortex, they are molecularly defined to exhibit differential axon extension specificity establishing at least two distinct cortical output projection circuits (cortico-brainstem vs. corticospinal) that might play distinct roles in skilled movement control. To our knowledge, Npy-Cre is the first molecular tool that specifically provides access to cortico-brainstem projections, distinguishing them from corticospinal projections, through development into maturity. This molecular access now enables investigations into the specific functional role(s) of cortico-brainstem neurons at maturity, which was previously not possible. Consequently, this developmental molecular delineation can now enable functional, circuit-level investigations at maturity. As proof of concept, we next tested the role of Npy^+^ CBN in skilled forelimb movement.

### Npy^+^ CBN are necessary for skilled forelimb motor control

Corticospinal circuits are widely recognized as the primary contributors to fine motor control of the forelimb, playing a crucial role in skilled movements via their connections to spinal motor neurons and interneurons in the cervical spinal cord^11^. However, recent research has also implicated brainstem-spinal circuits in skilled forelimb movements in mice^5,6,28^, suggesting that the brainstem, traditionally associated with more basic motor functions, might also serve as a key hub for executing complex motor tasks. Building on these findings, we hypothesized that Npy-expressing CBN during development, through innervation of brainstem structures, might play a critical role in skilled forelimb movements in adulthood, particularly in fine motor tasks such as reaching and grasping.

**Fig. 6:**
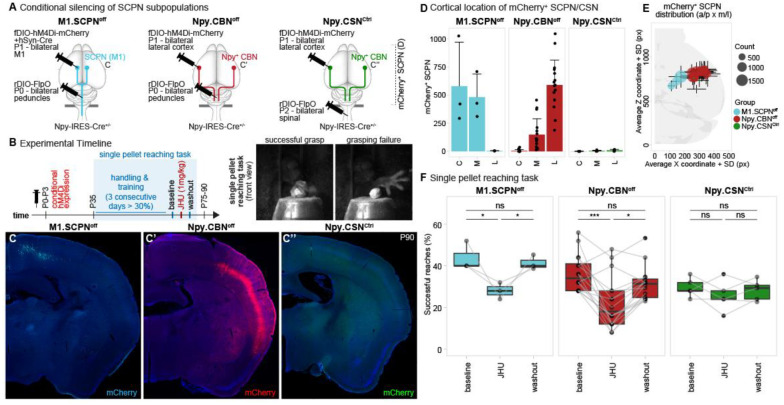
Selective silencing of Npy^+^ CBN leads to deficit in skilled movement task. (A) Schematic of conditional hM4Di expression in different SCPN subpopulations using intersectional viral labeling in Npy-Cre mice. In the M1.Ped control group (left panel), hM4Di-mCherry is targeted to M1 SCPN. In the Npy.Ped experimental group (middle panel), hM4Di-mCherry is targeted to lateral Npy^+^ CBN. In the Npy.SCc negative control group (right panel), hM4Di-mCherry is targeted to putative Npy^+^ CSN projecting to the spinal cord. (B) Timeline for behavioral analysis. hM4Di expression is induced at P1-P3. Mice are trained on the single pellet reaching task from P35 until they attain proficiency (>30% successful reaches on 3 consecutive days). Pellet reaches are then assessed on 3 consecutive days: baseline, during hM4Di ligand (JHU, 1 mg/kg) administration, and after washout (P75–90). (C) 3D reconstruction of mCherry^+^ SCPN in M1.Ped, Npy.Ped, and Npy.SCc groups. (D-D”) Coronal sections of injected mice at P90 showing mCherry (dsRed) immunofluorescence in M1.Ped (D), Npy.Ped (D’), and Npy.SCc (D”) groups, confirming successful targeting of distinct SCPN subpopulations in each group. (E) Quantification of mCherry^+^ SCPN in cingulate (C), medial (M), and lateral (L) cortex across groups. M1 SCPN (M1.Ped) are located in cingulate and medial cortex; Npy^+^ CBN (Npy.Ped) are predominantly in lateral cortex, with no Npy^+^ CSN labeled in Npy.SCc. (F) Distribution analysis of mCherry^+^ SCPN across anterior-posterior (a/p) and medial-lateral (m/l) axes shows predominant labeling of SCPN in medial cortex for M1.Ped and lateral cortex for Npy.Ped groups, indicating distinct SCPN subpopulation targeting. (G) Boxplots showing successful reaches (over 25 attempts) in the single pellet reaching task across baseline, JHU administration, and washout conditions. Silencing Npy^+^ CBN in the Npy.Ped group significantly reduces successful reaches comparable to M1.Ped control group, while targeting Npy^+^ CSN (Npy.SCc) shows no significant effect. Statistical significance is indicated with p-values.

To investigate the potential role of adult cortico-brainstem circuitry in skilled forelimb movement, we used the developmental expression of Npy by CBN to conditionally express inhibitory DREADDs in Npy-Cre mice during development to selectively silence this circuit in adulthood. For this, we used intersectional viral labeling in Npy-Cre mice to avoid labeling of Npy^+^ neurons in cortex or brainstem that are not Npy^+^ CBN. We injected a Cre-dependent retro-AAV-FlpO bilaterally into the cerebral peduncles at P0, followed by an injection of FlpO-dependent DREADD (AAV-fDIO-hm4Di-mCherry), into the lateral cortex at P1 (Fig. 6A). This strategy allowed us to target hM4Di-mCherry to Npy^+^ SCPN in Npy-Cre mice. To rule out functional effects mediated by any putative Npy^+^ CSN, a separate cohort of Npy-Cre mice received bilateral retro-AAV-FlpO injections in the cervical spinal cord. As a positive control, we also targeted hM4Di-mCherry to SCPN in primary motor cortex (M1). To activate DREADD expression by SCPN in this control group, we co-injected AAV-hSyn-Cre along with the hm4Di-mCherry into medial cortex, since Npy-Cre is not expressed by SCPN in medial cortex. We therefore generated the following three groups of mice: 1) M1.Ped (positive control group) where the hm4Di-mCherry is expressed by all SCPN in M1, 2) Npy.Ped experimental group in which the hm4Di-mCherry is specifically targeted to Npy^+^ CBN in lateral cortex, and 3) Npy.SCc negative control group to target hm4Di-mCherry expression in putative Npy^+^ CSN projecting to the spinal cord.

We analyzed the distribution of hm4Di-mCherry^+^ SCPN in all three groups. 3D reconstruction of hm4Di-mCherry^+^ SCPN at P90 confirmed accurate targeting to the respective subpopulations in each group (Fig. 6C, D–D”). Quantification of the cortical distribution of hm4Di-mCherry^+^ SCPN revealed patterns consistent with the expected distribution across the cingulate (C), medial (M), and lateral (L) cortex in the three groups: In the M1.Ped group, hM4Di-mCherry^+^ SCPN are distributed in the cingulate and medial cortex. In the Npy.Ped group, hM4Di-mCherry^+^ CBN are predominantly located in lateral cortex, with minimal overlap with SCPN in M1 (Fig. 6E, F). As expected, the Npy.SCc negative control group showed no labeling of hM4Di-mCherry^+^ CSN (Fig. 6E, F; Fig. 5C).

At P35, mice were trained on the single pellet reaching task until they reached proficiency (>30% successful reaches on 3 consecutive days). We then assessed reaching performance on 3 subsequent days – day 1) baseline; day 2) during hM4Di ligand (JHU) administration; and day 3) washout (Fig. 6B). As expected, the M1.Ped control group showed a significant reduction in successful reaches following hM4Di ligand administration to silence primary motor cortex (Fig. 6G, left panel, 44 % ± 6.9 at baseline, 28 % ± 4 after JHU administration)^11,29–32^. Given the absence of hm4Di-mCherry+ SCPN, the Npy.SCc group also did not show any deficits in reaching success (Fig. 6G, right panel, 29.6 % ± 4.6 at baseline, 25.6 % ± 7.2 after JHU administration). However, selective silencing of Npy^+^ CBN in the Npy.Ped group resulted in a significant reduction in reaching performance, as evidenced by a decrease in successful reaches (Fig. 6G, middle panel, 36.5 % ± 8.9 at baseline, 22.3 % ± 12.6 after JHU administration), highlighting a critical role for Npy^+^ CBN in skilled forelimb movement. Our results show that specific cortico-brainstem circuits are established early in development to support skilled forelimb function at maturity. Moreover, this underscores the potential of such developmental delineation to investigate the functional role(s) of these circuits in adulthood.

### Axon collateralization by Npy^+^ CBN vs. CSN in the brainstem differs along rostro-caudal axis

**Extended Data Fig. 6:**
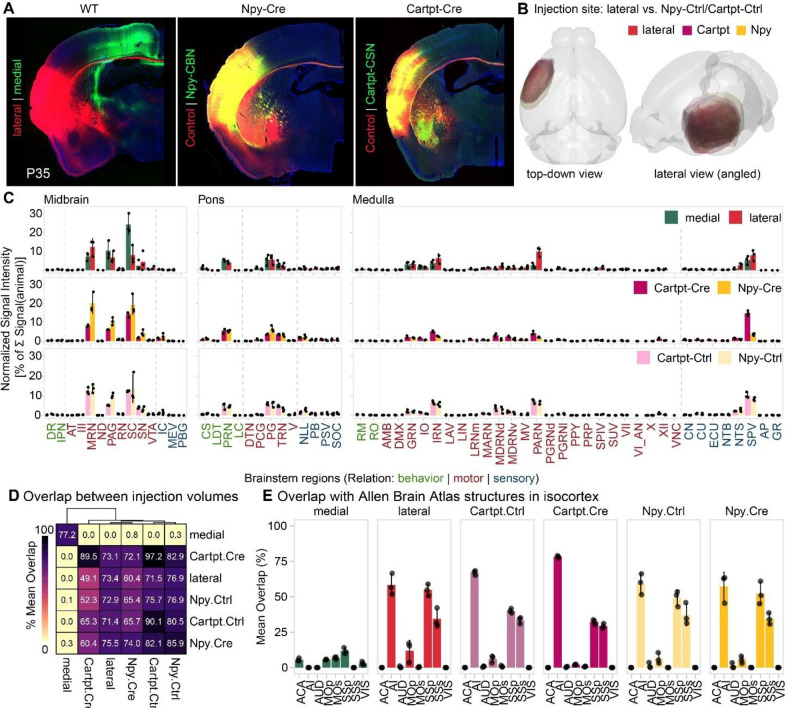
Consistent cortical injections for anterograde labeling enables downstream analysis of brainstem innervation by distinct SCPN subpopulations. (A) Representative cortical sections of P35 mouse brains displaying AAV-labeled cortical injection sites for downstream analyses of axon collateralization in the brainstem. (B) 3D rendering of all injected brains from WT, Npy-Cre and Cartpt-cre aligned to the CCFv3 reference atlas showing injection site in lateral cortex in of control AAV (constitutive, not Cre-dependent). The comparison shows alignment of injections across all three groups. (C) Quantification of SCPN axon collateralization in distinct brainstem regions, performed by measuring normalized signal intensity, comparing medial vs. lateral SCPN (top panel) and Npy+ vs. Cartpt+ SCPN (middle panel). Note that there is no difference in control labeling in Npy-Cre vs. Cartpt-Cre mice. The brainstem regions are shown along the x axis and are color coded as motor- (in red), sensory- (in blue), or behavior- (in green) related (annotations as described by the Allen Brain Atlas). (D) Correlation plot comparing injection volumes across all overlap analysis shown indicates no overlap between medial and other injections and a strong correlation between lateral and Cre mouse line injections. (E) Estimated overlap with selected Allen Brain Atlas structures highlights similarities between lateral and Cre injections, whereas medial injections remain mostly within MOp/MOs. Abbreviations for brainstem regions and cortical structures can be found in Supplementary Table 3 and Supplementary Table 4, respectively.

**Extended Data Fig. 7:**
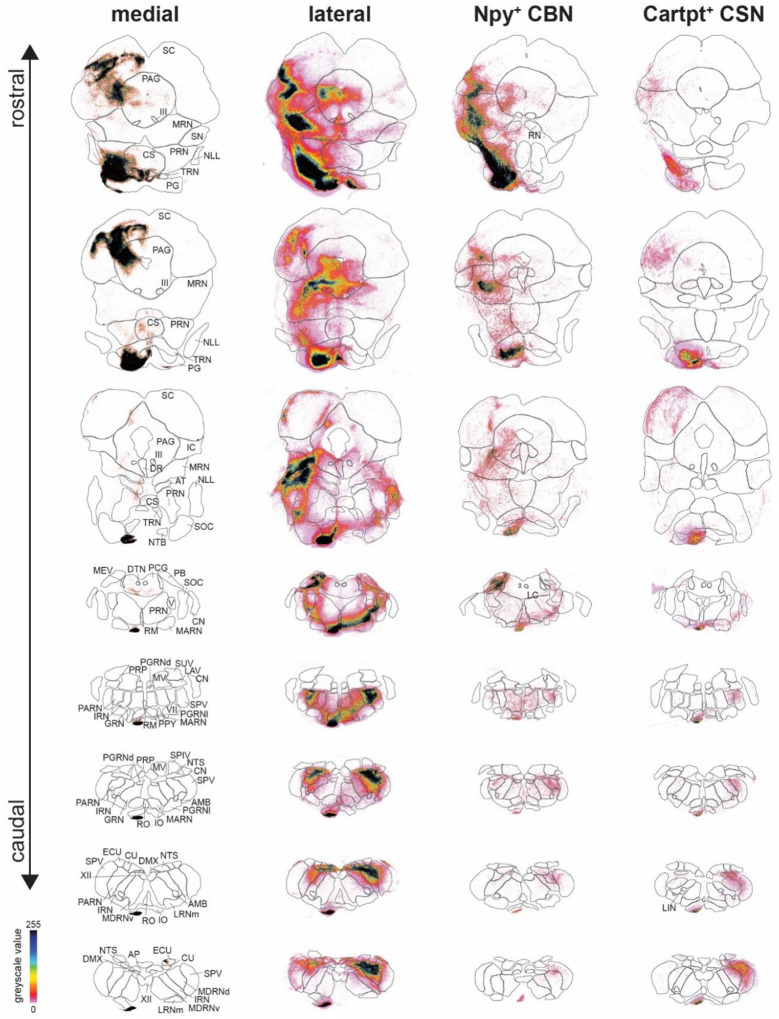
Differential brainstem innervation by distinct SCPN subpopulations. Rostro-caudal distribution of SCPN axonal projections in the brainstem color coded to reflect mean grey value (0 white, 255 black; see heatmap on lower left). Brainstem sections are organized from rostral (top) to caudal (bottom), with key anatomical landmarks detected using StARQ, our novel machine learning pipeline (key to the abbreviations for the different structures are in Supplementary Table 3). Note the differences between projections from medial vs. lateral SCPN subpopulations as well as between Npy^+^ CBN and Cartpt^+^ CSNc.

**Fig. 7:**
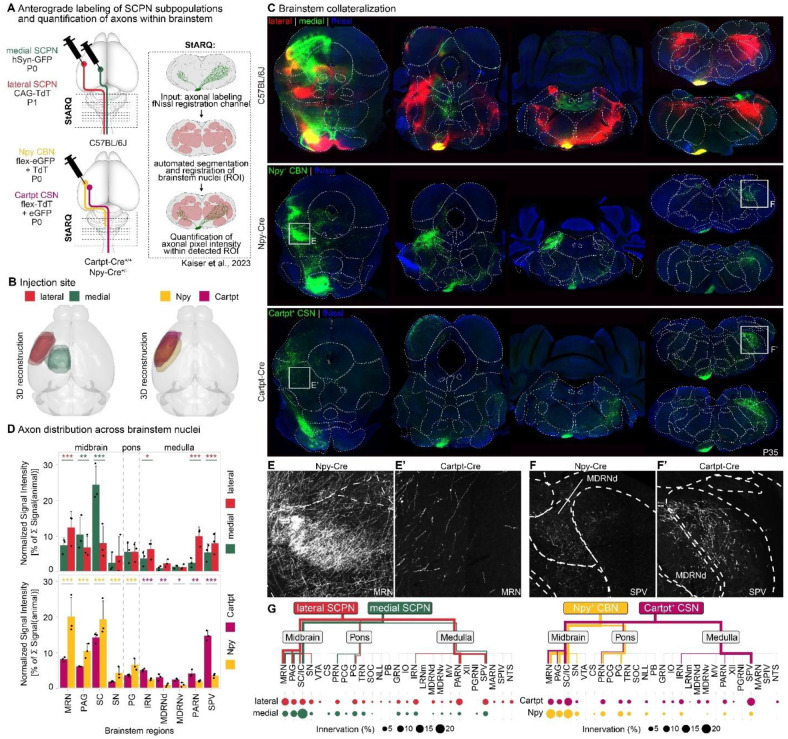
Distinct cortico-brainstem collateralization patterns of SCPN subpopulations reveal specialized connectivity. (A) Schematic of anterograde labeling strategy used to investigate brainstem collateralization by medial vs. lateral SCPN axons and Npy^+^ CBN vs. Cartpt^+^ CSNc axons. Coronal brainstem sections were analyzed using the StARQ^33^ machine-learning pipeline for automated segmentation and quantification of axon density within brainstem regions of interest (ROI). (B) 3D reconstruction of injection sites shows non-overlapping medial and lateral SCPN labeling, as well as distinct Npy-Cre and Cartpt-Cre labeling in the lateral cortex. (C) Representative brainstem sections show rostro-caudal distribution of axonal projections from medial and lateral SCPN subpopulations. Striking differences in collateralization patterns are observed within the brainstem between these subpopulations. Npy-Cre+ CBN and Cartpt-Cre+ CSN, both subsets of lateral SCPN, also display distinct rostro-caudal innervation patterns. (D) Quantification of axonal distribution across brainstem nuclei, comparing lateral vs. medial SCPN and Npy-Cre+ CBN vs. Cartpt-Cre+ CSN. Pixel intensity was normalized to the total signal within each animal. Only regions with significant differences are shown. (E, E’) Zoom-in on the MRN region reveals denser innervation by Npy-Cre+ CBN axons compared to Cartpt-Cre+ CSN. (F, F’) Zoom-in on the SPV and MDRNd regions demonstrates preferential innervation by Cartpt-Cre+ CSN axons. (G) Connectivity map depicting the collateralization of lateral vs. medial SCPN and the differential connectivity of Npy^+^ CBN vs. Cartpt^+^ CSN across brainstem regions. Regions with input >1% of signal are shown. Connectivity above 5% is represented through colored lines (colors indicate SCPN subpopulation), with line thickness corresponding to innervation strength (max. 20%). Bubble map shows innervation strength within each brainstem region for each group. Abbreviations are listed in Supplementary Table 3.

Given the role of Npy^+^ CBN in controlling skilled forelimb movement, we next sought to identify specific cortico-brainstem circuits underlying this function. Npy^+^ CBN reside in lateral cortex, including regions such as the sensorimotor and insular cortex but excluding the primary motor cortex. We thus first analyzed axon collateralization in the brainstem by all SCPN in lateral cortex and compared this with SCPN projections arising from medial cortex, i.e., cortical areas typically associated with motor control, such as the primary motor cortex^1,3^. We injected AAV-hSyn-GFP into medial and AAV-CAG-TdTomato into lateral cortex of *C57BL6* WT mice at P1 and analyzed axon collateralization at P35. We first confirmed that GFP and TdTomato reporter expression does not overlap in cortex using 3D reconstruction of the injection sites to ensure that we analyzed brainstem collateralization by SCPN residing in distinct cortical areas (Fig. 7B, Extended Data Fig. 6D): we confirmed that medial SCPN originate from areas such as cingulate and primary sensorimotor cortices (Extended Data Fig. 6E), and lateral SCPN from primary and secondary somatosensory cortex and insular cortex (Extended Data Fig. 6E). Our newly established machine learning-based segmentation and quantification pipeline allowed us to quantify axon collateralization across the rostrocaudal extent of the brainstem (midbrain, pons and medulla, Extended Data Fig. 6C, Extended Data Fig. 7)^33^.

We hypothesized that brainstem collateral distribution by lateral cortex SCPN would at least partially resemble the collateralization from primary motor cortex, given its role in skilled movement control. We find, however, striking differences in collateralization distribution along the rostro-caudal axis (Fig. 7C, D). Medial SCPN collaterals predominantly innervated superior colliculus (SC, 24.38 % ± 5.19 of the overall brainstem collaterals) and periaqueductal gray (PAG, 10.46 % ± 4.81). In contrast, lateral SCPN targeted similar areas but with more extensive projections along the rostro-caudal axis, including midbrain reticular nuclei (MRN, 12.43 % ± 4.46), PAG (6.85 % ± 3.10) and SC (8.07 % ± 4.76) in the midbrain, and intermediate reticular nuclei (IRN, 6.41 % ± 2.52), spinal nucleus of the trigeminal (SPV, 8.01 % ± 2.86) and parvicellular reticular nuclei (PARN, 9.99 % ± 2.56) in the medulla (Fig. 7C, D, G). Interestingly, even in brainstem nuclei receiving input from both medial and lateral SCPN, such as the SC, axon collaterals rarely overlap. Instead, their collateralization follows a somatotopic organization, with medial and lateral SCPN targeting distinct, spatially segregated regions within the nucleus (Fig. 7C, upper panel). Thus, even though both lateral and medial cortex SCPN function in controlling skilled forelimb movement, they exhibit striking differences in their brainstem innervation/collateralization (Extended Data Fig. 7).

Since Npy^+^ CBN reside interdigitated with Cartpt^+^ CSNc in lateral cortex, we next compared brainstem axon collateralization of these two distinct lateral SCPN subpopulations (Fig. 7A, lower panel). For this, we injected AAVs expressing Cre-dependent reporters into the lateral cortex of either Npy-Cre or Cartpt-Cre mice at P1 and analyzed the mice at P35. We first performed 3D reconstructions of the injection sites in cortex across all mice and confirmed successful targeting of both SCPN subpopulations in lateral cortex, with comparable injection sites across Allen Brain Atlas isocortex substructures (Fig. 7B, Extended Data Fig. 6A, B, E). Using the same machinelearning-based segmentation pipeline to quantify collateralization within brainstem regions (Fig. 7C – G), we find that Npy^+^ CBN projections are predominantly confined to midbrain motor regions like the MRN (20.10 % ± 5.33), SC (19.35 % ± 5.15) and PAG (10.54 % ± 2.03). These same areas are also targeted by Cartpt^+^ CSN, albeit to a lesser extent (MRN: 8.19 % ± 0.62, SC: 14.25 % ± 1.43, PAG: 6.16 % ± 0.05). However, Cartpt^+^ CSN additionally targeted more caudal regions within the medulla, including SPV (14.79 % ± 1.40), IRN (5.15 % ± 0.43) and PARN (4.23 % ± 1.01). Therefore, while both Npy^+^ CBN and Cartpt^+^ CSNc projections collectively resemble the overall collateralization by SCPN in lateral cortex, there is a clear distinction between the two SCPN subpopulations, with Npy^+^ CBN axons targeting more rostral areas and Cartpt^+^ CSN projecting more extensively into caudal regions (Fig. 7G). This suggests that Npy^+^ CBN may play a specialized role in modulating skilled movement by influencing rostral brainstem areas.

Overall, this study reveals novel molecular programs that delineate axon extension differences by SCPN subpopulations that go beyond areal controls. Further, these developmentally defined subpopulations of SCPN contribute to adult motor circuitry, specifically for skilled movements. Our findings highlight how early molecular specification drives SCPN diversification to support movement regulation and underscore the critical role of cortico-brainstem connectivity in skilled movement behaviors.

## Discussion

Skilled movement execution relies on precise connectivity between SCPN and their supraspinal as well as spinal targets. In this work, we present a new model for developmental diversification of SCPN connectivity, demonstrating how early molecular programs shape adult motor circuitry. We identify novel molecular signatures within SCPN that define previously unrecognized axon extension specificity between CBN and CSN at the brainstem-spinal transition zone. This molecular delineation is independent of areal specification. CBN restrict their axon extension to the brainstem and establish a direct cortico-brainstem pathway, while CSN extend axons past the brainstem into the spinal cord. We establish the first known molecular differences between these subpopulations at this critical developmental stage, with Npy specifically expressed in CBN and Cartpt in CSN. This early molecular delineation sets up the foundation for adult motor circuitry, ensuring the establishment of two anatomically segregated pathways that originate in the same cortical area. Notably, despite terminating in the brainstem, Npy^+^ CBN are indispensable for skilled forelimb movements and exhibit striking differences in brainstem innervation compared to CSN. These previously undescribed Npy^+^ CBN represent a novel component of motor circuitry, providing a new basis for understanding circuit and functional diversification. Together, our findings underscore how developmental molecular signatures diversify SCPN connectivity, establishing anatomically and functionally discrete circuits essential for skilled movements at maturity.

### Cortico-brainstem axons are limited to supraspinal levels from initial stages of axon extension, establishing a *de novo* cortico-brainstem circuit

Seminal work in the field has shown that all SCPN initially extend exuberant axonal projections to the spinal cord early in development, and that specificity of connectivity is achieved through selective axonal pruning^14–16,34,35^. These investigations have established that at least a subset of cortico-brainstem connectivity emerges as a result of selective axonal pruning of CSN axons that initially project to the spinal cord. Molecular factors play a critical role in this refinement process, and specific genes regulating axonal pruning have been identified^17,18^.

However, our findings reveal an additional, previously unrecognized developmental process that specifies this cortico-brainstem connectivity. Using retrograde labeling from the earliest stages of axon extension, we show that a subset of CBN restrict their axons to supraspinal levels and do not even transiently extend axons into the spinal cord, highlighting that at least a subset of cortico-brainstem circuitry is established *de novo*. This specificity is established before any refinement occurs via pruning, indicating that not all SCPN follow the same initial trajectory of axon extension into the spinal cord. Similar developmental axon extension specificity is observed at the cervical-thoracic transition zone, distinguishing CSN targeting cervical vs. thoraco-lumbar spinal segments^20^ and is molecularly controlled via differential gene expression between these subpopulations^19^. This suggests that the brainstem-spinal transition zone represents another anatomical site for regulating SCPN axon extension specificity, driven by molecular programs that establish CBN projections during development via regulation of axon extension.

Notably, adult scRNA-seq studies have identified molecular differences between spinal projecting neurons across different brain regions^36^ and specifically CSN projecting to cervical vs. lumbar spinal cord^37^. However, these adult molecular signatures are absent from our developmental dataset, and conversely, our developmental molecular signatures do not map to these adult neurons (data not shown). These results underscore that developmental mechanisms governing SCPN axon extension specificity are transient and do not persist into adulthood. Instead, early molecular regulators direct SCPN axons to their appropriate segmental levels, establishing a blueprint for circuit formation that ensures precise connectivity and functional specialization later in life.

### Molecular diversification of SCPN connectivity integrates axon extension specificity with cortical location and temporal code of differentiation rates

Cortical location has long been known to underlie diversification of SCPN connectivity. Molecular regulators have been identified that distinguish SCPN in distinct cortical areas and function to control their distinct subcerebral connectivity^18,38–41^. SCPN in motor versus visual cortical areas express distinct genes that control their differential connectivity via pruning in an area-dependent manner^16,17,40^. More recently, such area-distinct genes have been identified that can be used to reprogram SCPN in motor cortex to prune their projections to more proximal targets, demonstrating the plasticity of their connectivity^18^. Further, this areal axis of SCPN molecular diversity is orthogonal to the temporal axis underlying their differentiation over time^18^. Our results establish that axon extension specificity represents yet another axis of SCPN molecular diversity in development.

Our molecular annotations show that the axis distinguishing SCPN by areal location into medial versus lateral SCPN is orthogonal to the axis of axon extension specificity. Further, the molecular diversity within medial and lateral SCPN clusters correlates with their diversity of projection targets. Lateral SCPN are relatively homogeneous, extending axons primarily to the brainstem and cervical spinal cord, while medial SCPN project to multiple levels across the neuraxis (brainstem, as well as cervical, thoracic, lumbar cord). In line with this, we find greater diversity in medial than lateral SCPN. Lateral SCPN, primarily from somatosensory and insular cortex, consist mostly of CBN, with a smaller subset of cervical-projecting CSN, consistent with our previous findings^20^. We find two molecularly distinct clusters in lateral cortex that represent this difference in axon extension: one for CBN (B.lat) and one for cervical-projecting CSN (S.lat). On the other hand, SCPN in medial cortex, encompassing primary and secondary motor cortex, exhibit greater molecular diversity likely reflecting their wider array of projection targets. Crim1 expression in our medial cluster (S.med) aligns with its previous identification as a marker of SCPN in primary motor cortex^18^, specifically identifying thoraco-lumbar projecting CSN within the broad population of medial SCPN^20^. Additional medial spinal clusters (S.med1, S.med2) likely represent further diversity within cervical-projecting CSN. Future investigations will identify and validate this developmental molecular delineation.

Temporal differentiation also plays a critical role in SCPN diversification and areal regulators can regulate the timing of molecular differentiation thereby regulating connectivity and function^42^. It is possible that the timing of differentiation controls the binary molecular and anatomical divergence between Npy^+^ CBN and Cartpt^+^ CSNc. There is precedence for such divergence being governed by developmental timing^43^. Intra-cortical projection neurons (ICPN) in primary somatosensory cortex can project either to primary motor or secondary somatosensory cortex, and their differential connectivity is controlled by regulating the pace of their differentiation. Intriguingly and in line with this, we find that Npy^+^ CBN are more differentiated than other SCPN subpopulations in early postnatal cortex. Additionally, in the developing spinal cord, long-range projection neurons are born earlier than local interneurons and maintain distinct molecular identities from E12.5 into adulthood^44^. Given that CBN project to more proximal targets than CSNc, similar temporally controlled mechanisms may contribute to their molecular, and eventually anatomical, distinction. These novel molecular delineators offer new opportunities to explore such timing-based differences in SCPN generation and differentiation during cortical development.

While temporal differentiation and cortical areal location guide early SCPN development, our findings identify a third, independent axis of SCPN diversification: axon extension specificity. From the earliest stages of development, CBN and CSN exhibit distinct axon extension trajectories: CBN restrict their axon extension within the brainstem, while CSN extend axons past the brainstem into the spinal cord. This distinction occurs within the same cortical area (e.g., within lateral cortex), highlighting that axon extension specificity operates independently of areal differentiation. This convergence of temporal differentiation, cortical location, and axon extension specificity ensures that SCPN within the same cortical area are set up early in development for distinct projection targeting at maturity. Thus, axon extension specificity plays a pivotal role in establishing anatomically and functionally discrete cortical output circuits.

### Molecular control over developmental SCPN axon extension

Given the importance of axon extension specificity in establishing SCPN connectivity and functional motor circuits, understanding the molecular mechanisms that direct SCPN axons to their eventual targets remains a critical area of research. Although scRNA-Seq has greatly enhanced our understanding of cell type diversity in both developing and adult cortex^23,24,26,45–52^, minority neuron populations such as SCPN are often underrepresented in these investigations. While these large-scale datasets are beginning to establish the developmental trajectories directing differentiation of distinct neocortical neuron subtypes^26,53,54^, they often fail to capture the molecular complexity of smaller populations such as SCPN, which comprise just 2–3% of all cortical neurons. As a result, these datasets lack the granularity needed to identify the molecular delineators driving SCPN diversification. Identifying such molecular complexity within these broad subtypes requires their enrichment for more in-depth analyses.

Previous work, using enrichment techniques has identified molecular mechanisms that distinguish SCPN from other neocortical projection neurons and control SCPN axon targeting toward subcerebral targets^3,39,55,56^. However, even with such enrichment approaches, our results show that investigating the molecular control over axon extension at specific levels of the neuraxis requires differential anatomical labeling at critical developmental times followed by appropriate integration of scRNAseq datasets. To date, scRNAseq approaches have not been used to directly investigate differential axon extension at any given level of the neuraxis. In our experimental approach, we could establish these molecular correlates of axon extension by performing retrograde labeling from two different rostro-caudal levels. Neurons labeled from the more rostral level (from the cerebral peduncles) represent the broader population, while the neurons labeled from the more caudal level (in the cervical cord) comprised a subset of this overall population. Using this subsetting approach in the scRNAseq integration, we uncovered molecular differences between CBN and CSN that otherwise reside in the same overall cortical location and even extend axons along the same white matter tract. Beyond identifying these molecular correlates, this approach also enabled prospective identification of these subpopulations at P0/P1, prior to the emergence of these anatomical differences in axon extension. Our results provide a proof-of-concept that integrating scRNAseq with retrograde labeling from different rostro-caudal levels at critical developmental stages can be more broadly applied to other long-range projection neurons to identify molecular regulators of axon extension during development.

We identify differentially expressed genes between CBN and CSN at a time when SCPN axon extension differences are established at the brainstem-spinal transition zone. *Cartpt* expression by CSNc increases from P1 to P7, aligning with the period of active CSN axon extension into the cervical spinal cord, suggesting that Cartpt might control CSNc axon extension. In contrast, *Npy* expression by CBN peaks earlier, possibly playing an equivalent or similar role in limiting CBN axon extension to the brainstem. Strikingly, we find genes with known functions in axon guidance that are differentially expressed between these two subpopulations at this early developmental time. These include Sema3e^57,58^ expressed by CBN, as well as Alcam^59,60^ expressed by CSNc. While some of these genes might regulate axon extension, they might also regulate subsequent steps in the establishment and/or refinement of corticospinal and cortico-brainstem circuitry. For instance, the proteoglycan Lumican does not control SCPN axon extension, but non-cell-autonomously regulates CSN axon collateralization in cervical gray matter^61^. In line with this, we find that CSNc specifically express Lrrtm3, which contains leucine rich repeats, similar to Lumican, and is a known regulator of synapse formation and differentiation^62,63^. Using our newly established molecular tools for distinguishing CBN from CSNc, the potential functions of these candidate genes and the molecular control over SCPN axon extension at the brainstem-spinal transition zone can now be investigated with precision. This will, in turn, provide new insights into the molecular mechanisms governing early development of neocortical connectivity with subcerebral targets.

### Developmentally established *de novo* cortico-brainstem circuit controls skilled forelimb movement in adulthood

The corticospinal tract has been extensively studied, providing a detailed understanding of its role in skilled movement^1,11,35,64,64–72^. In contrast, cortical inputs to the brainstem, particularly *de novo* cortico-brainstem pathways, remain less understood despite their potential to diversify motor control^73^, These pathways likely modulate complex motor outputs, complementing corticospinal functions by targeting distinct brainstem regions involved in movement coordination^5,6,9,74^. However, it has been a longstanding challenge to delineate the specific contributions of cortico-brainstem vs. corticospinal pathways to skilled movement since these neurons are interdigitated within cortex and both provide inputs into the brainstem—CBN directly via a de novo established cortico-brainstem circuit, and CSN via collateral branches of the corticospinal tract. The novel intersectional viral labeling and conditional silencing tools developed in this study enabled the selective targeting of Npy^+^ CBN, allowing us to overcome this barrier for the first time and investigate their distinct role in motor control.

SCPN from primary motor cortex have been well-characterized for their contributions to skilled movements^28,29,75,76^. Recent studies further highlight a topographic organization in anterior motor cortex projections, with medial SCPN projecting to ventral medulla to support reaching, and lateral SCPN axons targeting dorsal medulla for food handling^77^. Our findings extend this concept, showing that axon collaterals from medial SCPN (rostral motor cortex) are primarily restricted to rostral brainstem regions such as superior colliculus (SC) and midbrain reticular formation (MRN), which forms the rostralmost part of the reticular formation. This is in line with single cell reconstructions that have found limited axon collateralization by SCPN in primary motor cortex within the brainstem^7,8^. In contrast, lateral SCPN exhibit more widespread and diverse brainstem connectivity throughout the rostro-caudal axis, likely reflecting functional specialization between these subpopulations that reside in distinct cortical locations.

Within lateral SCPN subpopulations, Cartpt^+^ CSN target primarily caudal regions such as the SPV and medullary reticular formation which have been shown to be involved in skilled reaching^5,28^. Conversely, Npy^+^ CBN display a rostrally biased projection pattern that partially overlaps with medial SCPN targets: The MRN has previously been implicated in postural control and skilled movement coordination^78^. Additionally, Npy^+^ CBN also collateralize within SC, which plays an essential role in accurate forelimb reaching^79^ by controlling proximal limb musculature^80,81^. This suggests the SC may serve as a relay through which CBN exert influence on skilled forelimb control. Interestingly, we find somatotopic differences in SC innervation both between medial and lateral SCPN, as well as within lateral SCPN subpopulations CBN and CSN, which potentially suggests specialized function for each of these subpopulations. While the SC has been implicated in skilled reaching, the specific functions of cortical inputs—particularly from CBN—remain to be fully elucidated. Future studies using our novel molecular tools will serve to address these questions.

Silencing of CBN leads to deficits in a reaching and grasping task, demonstrating their indispensable role in skilled movement, similar to CSN^11,29–32^. While most research has focused on corticobulbar projections from primary motor cortex^7,28,75,76^, the Npy^+^ CBN studied here are located in the lateral cortex, traditionally associated with sensory processing. This raises questions about their role in sensory modulation, motor control, or both. Prior studies have shown that primary somatosensory cortex (S1) plays a significant role in skilled movement regulation^82^, with CSN in S1 contributing to this process^11^. Our findings reveal that Npy^+^ CBN, despite residing in a sensory-associated cortical area, are indispensable for skilled forelimb movement. The role of Npy^+^ CBN in skilled forelimb movement could be via direct innervation of motor control centers in the brainstem or by regulation of sensory modulation. Notably, the corticospinal tract has been associated with both motor control and sensory modulation. CSN are known to control motor outputs via their synaptic targets in the spinal cord that ultimately provide cortical control over both spinal motor ouputs as well as sensory modulation^11,83^. Npy^+^ CBN reside in cortical areas with known projections to brainstem regions previously associated with tactile feedback^9^, suggesting a potential role in sensory modulation. In addition, Npy^+^ CBN also target premotor nuclei crucial for skilled movement which could hint at direct involvement in motor circuits^28^. Thus, CBN anatomy is consistent with influences on both sensory and motor pathways. The distinction between sensory modulation and motor control roles remains to be fully elucidated, but the broad innervation and functional importance of CBN suggest they play a critical role in both domains.

Collectively, these results underscore the essential role of these developmentally defined circuits within the SCPN connectome in execution of skilled motor control in the adult. Lateral Npy^+^ CBN provide a previously unrecognized layer of motor control. Their unique rostral brainstem targeting and integration into brainstem circuits highlight the diversity and specialization of SCPN subpopulations. These findings emphasize that skilled motor behaviors rely on more than the primary motor cortex; lateral cortical regions and their distinct contributions are equally indispensable. Further research into CBN’s dual sensory and motor roles will be critical for understanding how these circuits support complex motor tasks in the adult nervous system. The tools developed in this study will facilitate the dissection of specific contributions from both cortico-brainstem and corticospinal pathways to skilled forelimb movements.

### CBN and CSN Pathways: Implications for Motor Control and Neurological Disorders

Investigating the fundamental mechanisms by which cortico-brainstem and corticospinal circuits are established during development provides critical insight into their potentially distinct functional contributions in different behaviors. Cortico-brainstem circuits, in particular, may support a diverse range of motor functions beyond limb movement, including orofacial behaviors, respiration, and posture^73,84,85^. The differences between cortico-brainstem and corticospinal circuits also have significant implications for a vast array of neurological disorders affecting motor ability. Plasticity of connectivity between the cortex and brainstem has been shown to contribute to movement recovery after stroke in both animal studies^12,86,87^ and in human subjects^88^. Whether this plasticity occurs exclusively in CBN or CSN or in both subpopulations is unknown. Therefore, it also remains unclear to what extent cortico-brainstem versus corticospinal connections contribute to this recovery. Our new molecular tools to investigate this newly described direct cortico-brainstem circuit will likely uncover novel mechanisms of motor control and recovery. Such findings could have implications for compensatory recovery or the progression of motor deficits in several other neurological disorders such as stroke, spinal cord injury, Parkinson’s disease, and amyotrophic lateral sclerosis^12,13,89–93^. The inherent plasticity and adaptability of these pathways – which may differ between CBN and CSN – also hold promise for therapeutic strategies, whether through promoting recovery of impaired motor skills, maintaining motor function in degenerative conditions, or enhancing rehabilitation outcomes^86,87,94^. However, much remains to be learned about how specific SCPN subpopulations uniquely contribute to these processes. By uncovering the molecular and developmental foundations of these pathways, future research can address both physiological motor functions and the broad spectrum of diseases that disrupt them, paving the way for more effective interventions and preventative measures.

In conclusion, the identification of distinct molecular programs that drive SCPN diversification provides critical insights into the developmental mechanisms that shape adult motor circuitry. By demonstrating that cortico-brainstem connectivity arises from an early, molecularly specified pathway—rather than solely through postnatal axonal pruning—we redefine the foundational principles of SCPN development. Npy^+^ CBN as a distinct SCPN subpopulation are essential for skilled forelimb control, which highlights an unrecognized layer of complexity in the organization and function of motor control circuits. These findings not only refine our understanding of cortical output organization but also offer novel targets for therapeutic intervention in movement disorders. By elucidating the molecular and anatomical frameworks underlying SCPN connectivity, this work establishes a foundation for future research aimed at precise modulation of these circuits to enhance recovery and functional outcomes in neurological injury and disease.

## Methods

### Data reporting

No statistical methods were used to predetermine sample size. The experiments were not randomized. Investigators were blinded to group allocation during behavioral experiments and outcome assessments wherever possible.

### Animals

All mouse studies were performed in accordance with institutional and federal guidelines and were approved by the Weill Cornell Medical College Institutional animal care and use committee. Wild-type CD1 and C57BL/6J mice were obtained from Charles River Laboratories (Wilmington, MA). The day of birth was designated as postnatal day 0 (P0). The following mouse lines were obtained from Jackson Laboratories: Npy-Cre (B6.Cg-Npytm1(cre)Zman/J, Stock No.: 027851^95^), Cartpt-Cre (B6;129S-Cartpttm1.1(cre)Hze/J, Stock No.: 028533), and Npy-Flpo (B6.Cg-Npytm1.1(flpo)Hze/J, Stock No.: 030211^96^). The Npy-FlpO;Cartpt-Cre line was established by breeding Npy-FlpO with Cartpt-Cre mice. Mouse lines were bred to heterozygosity with wild-type C57BL/6J mice, except for Cartpt-Cre, which was bred to homozygosity. Animals were housed in groups of four to five under a constant 12 h light/dark cycle with food and water *ad libitum*.

### Retrograde labeling of SCPN subpopulations

Retrograde labeling from the cerebral peduncles or cervical spinal cord was performed using a pulled glass micropipette attached to a nanojector (Nanoject II/III, Drummond Scientific, Broomall, PA) at a rate of 23 nl/second as previously described^19,20,97^. Cholera Toxin B subunit (CTB; Thermo Scientific) was injected bilaterally at 3 injection sites at the cerebral peduncles (161nl injected at each site in 23nl increments; total of 483 nl) or into the spinal segment C1/C2 (both sides of the midline, total of 207 nl), respectively, under ultrasound-guided backscatter microscopy (Vevo 2100; VisualSonics, Toronto, Canada). Injections were performed at the time point of initial axon extension (P0 at cerebral peduncles, P2 at cervical spinal cord^22^) unless stated otherwise.

For AAV-mediated retrograde labeling of Npy^+^ or Cartpt^+^ SCPN in their respective Cre reporter mouse lines, rAAV-flex-eGFP (2.3 × 10^13^ GC/mL; Addgene, 51502-AAVrg^98^) and rAAV-CAG-tdTomato (2.5 × 10^13^ GC/mL; Addgene, 59462-AAVrg) were co-injected into the cerebral peduncles at P0 in Npy-IRES-Cre or Cartpt-IRES-Cre mice. For AAV-mediated retrograde labeling of Npy^+^ or Cartpt^+^ CSN, rAAV-flex-eGFP and rAAV-CAG-tdTomato were co-injected into the cervical spinal cord level C2 at P3 in Npy-Cre or Cartpt-Cre mice respectively. The AAV pCAG-FLEX-EGFP-WPRE was a gift from Hongkui Zeng (Addgene viral prep # 51502-AAVrg; http://n2t.net/addgene:51502; RRID:Addgene_51502) and AAV-CAG-tdTomato (codon diversified) was a gift from Edward Boyden (Addgene viral prep # 59462-AAVrg; http://n2t.net/addgene:59462; RRID:Addgene_59462).

For dual retrograde labeling of Npy^+^ and Cartpt^+^ SCPN, rAAV-flex-eGFP (2.3 × 10^13^ GC/mL; Addgene, 51502-AAVrg) and rAAV-fDIO-mCherry (2.2 × 10^13^ GC/mL; Addgene, 114471-AAVrg) were co-injected into the cerebral peduncles at P0 in Npy-Flpo;Cartpt-Cre double transgenic mice. The pAAV-Ef1a-fDIO mCherry was a gift from Karl Deisseroth (Addgene viral prep # 114471-AAVrg; http://n2t.net/addgene:114471; RRID:Addgene_114471).

### Anterograde labeling of SCPN subpopulations

For AAV-mediated anterograde labeling of Npy^+^ or Cartpt^+^ SCPN in Cre mice, Cre-dependent reporter AAV ((AAV1-flex-GFP (2 × 10^13^ GC/mL; Addgene, 51502-AAV1) or AAV1-flex-tdTomato (2.6 × 10^13^ GC/mL; Addgene, 28306-AAV1)) was co-injected with a control reporter AAV ((AAV1-CAG-tdTomato (1.5 × 10^13^ GC/mL; Addgene, 59462-AAV1) or AAV1-hSYN-eGFP (2.7 × 10^13^ GC/mL; Addgene, 50465-AAV1)) at P0 into rostro-lateral cortex (345 nl). AAV was injected unilaterally under ultrasound-guided backscatter microscopy via a pulled glass micropipette attached to a nanojector at a rate of 23 nl/second. The pAAV-FLEX-tdTomato was a gift from Edward Boyden (Addgene viral prep # 28306-AAV1; http://n2t.net/addgene:28306; RRID:Addgene_28306) and the pAAV-hSyn-EGFP was a gift from Bryan Roth (Addgene viral prep # 50465-AAV1; http://n2t.net/addgene:50465; RRID:Addgene_50465).

For dual AAV-mediated anterograde labeling of Npy^+^ and Cartpt^+^ SCPN in Npy-Flpo;Cartpt-Cre mice, AAV1-flex-eGFP and AAV1-fDIO-tdTomato (2.2 × 10^13^ GC/mL; Addgene, 128434-AAV1) were co-injected at P1 into rostro-lateral cortex (345 nl). The AAV-Ef1a-fDIO-tdTomato was a gift from Patricia Jensen (Addgene viral prep # 128434-AAV1; http://n2t.net/addgene:128434; RRID:Addgene_128434).

### Fluorescent activated cell sorting (FACS) of whole cells

Brains of retrogradely labeled mice were collected at P1 (SCPN group) or P3 (SCPN or CSN group). Tissue was kept in cold buffers and on ice at all times. Brains were rapidly dissected and sliced into 800 μm coronal sections using a TC-1 Tissue Chopper (Electron Microscopy Sciences). Sections containing labeled cells were selected under a fluorescent dissection scope (Nikon SMZ18), and the entire mediolateral extent of sensorimotor cortex was collected and pooled across the litter (8–10 brains / sample). A single cell suspension of the samples was obtained using enzymatic (15min, 37°C) and mechanical dissociation (gentle trituration using rounded glass Pasteur pipettes of decreasing diameter (600um, 300um)) followed by filtration through a 40μm mesh (Biologix, 15–1040-1). SCPN were FACS-purified from the single cell suspension using a WOLF flow sorter (NanoCellect Biomedical) with standard settings and an adapted threshold for cell size (30000). To enhance sorting efficiency and reduce contamination, cells were first sorted at a high concentration (~6 × 10^6^ cells/ml), followed by a secondary sort of the initially sorted cells at a lower concentration (~2 × 10^5^ cells/ml). This two-step process enabled efficient enrichment of CTB 555-labeled cells. We collected ~15000 cells in 5ml per sort. Cells were enriched for downstream processing by centrifugation at 80g for 10 minutes and immediately prepared for single-cell RNA sequencing.

### Single-cell RNA sequencing

Each sample (P1.SCPN, P3.SCPN, and P3.CSN) was sequenced using the Chromium 10x Single Cell 3’ pipeline, following the standard protocol. For SCPN samples, experiments were performed on two separate occasions (biological replicates), with two technical replicates collected each time. For CSN samples, two technical replicates were collected. Briefly, single-cell suspensions of 5,000 to 10,000 cells were loaded onto a Chromium Chip B/G and processed using the standard protocol for the Chromium Next GEM Single Cell 3ʹ GEM and Gel Bead Kits v3/v3.1. Libraries were prepared with the Chromium Next GEM Single Cell 3ʹ Library Kit v3.1 and Chromium i7 Multiplex barcodes. The sequencing libraries (n=11; P3.CSN (2), P3.SCPN (5), P1.SCPN (4)) were assessed for quality on the Agilent TapeStation (Agilent Technologies, Palo Alto, CA, USA), quantified with a Qubit 2.0 Fluorometer (Invitrogen, Carlsbad, CA), and pooled libraries were quantified by qPCR (Applied Biosystems, Carlsbad, CA, USA). The pooled libraries were clustered on 5 lanes of a flow cell and loaded onto an Illumina HiSeq instrument (4000 or equivalent) according to the manufacturer’s instructions, sequenced in a 2×150bp configuration with 8 bp single indexing as recommended by 10X Genomics.

### Perfusion, fixation and tissue processing.

For labeling studies, mice were terminally anesthetized using ice (mice younger than P7) or a mix of ketamine (150 mg/kg; Covetrus, NDC 11695–0703-1) and xylazine (15 mg/kg %, Akorn, Inc., NDC 59399–111-50) (mice older than P7). Animals were transcardially perfused with 10ml PBS followed by 10ml cold 4% paraformaldehyde (PFA). Brains were dissected, post-fixed in the same solution overnight and stored at 4°C in PBS until further use. After cryopreservation in 30% sucrose overnight, tissues were directly embedded in Tissue-Tek OCT Compound and cut into coronal sections (50 μm, 6 series collected) on a cryostat (Leica CM1860). Free-floating sections were collected and stored at 4°C in PBS until further processing. For *in situ* hybridization, brains were sectioned at 30 μm and collected on-slide across 12–14 slides. Slides were stored at −20°C and processed further the following day.

### Immunolabeling of SCPN subpopulations and axons

To visualize cell bodies of retrogradely labeled Cartpt^+^ and Npy^+^ SCPN in the cortex, free floating 50 μm cortex sections were blocked in a BSA blocking solution (0.3% BSA in PBS with 0.3% Triton-X) for 30 minutes at room temperature. Sections were incubated in rabbit anti-GFP, 1:500 (Invitrogen, A-11122) overnight at 4°C. Secondary antibody goat anti-rabbit Alexa Fluor 647 (Invitrogen, A-21245) was used at a dilution of 1:250 and sections were incubated for 3 hours at room temperature. Following secondary antibody incubation, sections were stained with DAPI, mounted on slide and cover slipped with Fluoromount-G (Invitrogen, 00–4958-02). Spinal sections (50 μm, axial) were processed using the same staining method to visualize axon tracts.

Following conditional silencing of SCPN subpopulations using inhibitory DREADDs, primary antibody Living Colors^®^ DsRed Polyclonal Antibody (Takara Bio USA, 632496) was used followed by secondary antibody goat ant-rabbit Alexa Fluor 546 (Invitrogen, A-11035) to label Npy^+^ CBN and putative Npy^+^ CSN, using staining protocol described above.

To visualize brainstem collaterals following anterograde labeling of Npy^+^ CBN or Cartpt^+^ CSN in Cre mice, free floating 50 μm brainstem sections were blocked with Image-iT FX Signal Enhancer (Invitrogen, I36933) as per manufacturer’s instructions. Standard staining protocol was subsequently followed. Primary antibody rabbit anti-GFP, 1:500 (Invitrogen, A-11122) was used to label Npy^+^ axons and rabbit anti-RFP, 1:500 (Rockland Immunochemicals, 600–401-379) was used to label Cartpt^+^ axons. NeuroTrace^™^ 500/525 Green (Invitrogen, N21480) or NeuroTrace^™^ 640/660 Deep-Red (Invitrogen, N21483) Fluorescent Nissl Stain was used to label neuronal cells as per manufacturer’s instructions. Sections were then mounted on slide and cover slipped with Fluoromount-G.

### *In Situ* Hybridization

Coronal sections were processed with the RNAScope Multiplex Fluorescent v2 kit (Advanced Cell Diagnostics, 323100). The probes used were: *Mm*-Npy (313321-C3), *Mm*-Cartpt (432001-C2), *Mm*-Alcam (462061-C3), *Mm*-Lrrtm3 (461501-C3), *Mm*-Klhl14 (510591), *Mm*-Sema3e (449631-C2) (Advanced Cell Diagnostics). The CTB signal in retrogradely labeled cells was amplified by immunofluorescence using a rabbit anti-CTB primary antibody (Abcam, ab34992, 1:200). The RNA-protein co-detection workflow was performed with the RNA-Protein Co-detection Ancillary Kit (Advanced Cell Diagnostics, 323180) according to the manufacturer’s instructions.

### Microscopy and image preprocessing

Imaging was performed using multiple microscopy setups depending on the sample type. The P4 coronal brain sections shown in Figure 1 (50 μm) were imaged in 4 μm z-stacks at 20x magnification on a Zeiss Axio Imager M2 microscope using Stereo Investigator software (MBF Biosciences), and single-plane images were extracted from the z-stacks using the Deep Focus tool in Neurolucida. Otherwise, coronal brain sections, spanning the cortex and the brainstem, were imaged at 10x magnification on the same microscope, with images processed as single TIF files in Neurolucida software (MBF Biosciences). RNAScope samples were imaged at 20x magnification on a Nikon A1R confocal microscope, with 5 z-stacks taken at 1 μm intervals around the focal point. Whole-mount overviews of the brain (dorsal and ventral view) were captured using a Nikon SMZ18 fluorescent dissection microscope.

### Tissue Preservation, Clearing, Immunolabeling and Imaging

A whole brain sample with dual retrogradely labeled Npy^+^ and Cartpt^+^ SCPN (Npy-FlpO;Cartpt-Cre) was cleared, stained, and imaged on a light sheet microscope by LifeCanvas Technologies (Cambridge, MA). The PFA-fixed brain was collected using the same protocols as described above. The brain was preserved using SHIELD reagents following the manufacturer’s instructions^99^ and delipidated with Clear+ delipidation reagents (LifeCanvas Technologies). After delipidation, the sample was immunolabeled with anti-GFP (6 μg; EnCor Biotechnology Inc.), anti-RFP (6 μg; Rockland Immunochemicals Inc.), and anti-NeuN (10 μg; EnCor Biotechnology Inc.) using eFLASH technology^100^, which integrates stochastic electrotransport^101^ and SWITCH^102^ on a SmartBatch+ device (LifeCanvas Technologies). For refractive index matching, the sample was incubated in 50% EasyIndex (RI = 1.52) overnight at 37°C, followed by 100% EasyIndex for one day. Imaging was performed using a SmartSPIM axially swept light sheet microscope with a 3.6x objective (0.2 NA). The resulting TIFF image stacks were converted to Imaris files (.ims) using the Imaris File Converter, and 3D reconstructions were created using Imaris software (Oxford Instruments).

For visualizing the brainstem and spinal cord in anterogradely labeled Npy^+^ and Cartpt^+^ SCPN (Npy-FlpO;Cartpt-Cre mice), a similar clearing procedure was performed in house using the SmartBatch+ active electrophoretic system (LifeCanvas Technologies). The whole brain (including spinal cord) was initially treated in SHIELD OFF for 3 days at 4°C, followed by SHIELD ON at 37°C for 24 hours. After an initial incubation in Delipidation Buffer for 24 hours, the sample underwent active delipidation for 30 hours following standard protocols. Index matching was achieved with 24 hours of 50% EasyIndex at 37°C, followed by 100% EasyIndex. The spinal cord was separately embedded in 1.5% 88°C melting point agarose (R2801, Thermoscientific) and subjected to repeated index matching within the agarose block. No additional antibody staining was applied for endogenous fluorescent proteins. Imaging was conducted on a Zeiss Lightsheet 7 (Carl Zeiss AG, Germany) using Zen Black (version 3.1), with samples immersed in low-fluorescence refractive index matching immersion oil (RI = 1.52, LDF, Cargille). Brainstem and spinal cord samples were imaged in dual channels (488, 561) at 5x magnification (0.94 × 0.94 × 3.5 μm), while whole brains were imaged at 2.5x magnification (1.83 × 1.83 × 5.23 μm). The images, captured as 16-bit CZI files, were stitched using Zen Blue (version 3.4.91). 3D reconstructions and video renderings were created in syGlass (version 1.8.2), utilizing the cut tool to remove excess imaging volume.

### Quantification of retrogradely labeled SCPN

At P4, due to the lack of available reference atlases, coronal sections were selected at four pre-defined bregma levels (1.95mm, 2.67mm, 3.27mm, 4.23mm to Bregma) for manual counting of retrogradely labeled SCPN/CSN using Neurolucida software (MBF Biosciences). To estimate cell distribution along the mediolateral axis, each cortical hemisphere was divided into 5 bins of equal width. We then quantified number of labeled neurons in the cingulate cortex (most medial bin), medial cortex (next 2 bins), and lateral cortex (outer/lateral most 2 bins). For in situ hybridization analysis, CTB^+^ SCPN/CSN co-labeled with Npy or Cartpt signals were manually selected using FIJI/ImageJ (version 1.54f^103^) and categorized into these mediolateral bins based on cell coordinate.

For P14 and P35 brains, AAV-labeled SCPN/CSN were manually selected in FIJI/ImageJ and transformed into the Common Coordinate Framework version 3 (CCFv3, 10 um atlas)^104^ using a custom MATLAB (The MathWorks) script based on the “AP_histology” package^105^. Cell coordinates were aligned to CCFv3 through affine transformations and their positions along the mediolateral axis (cingulate, medial, or lateral) were classified as described above. Data were visualized in 3D, with labeled cells color-coded by group. The mean rostro-caudal (X) and dorsolateral (Z) coordinates were calculated for each group, along with standard error, to represent cell distribution across these two axes.

### 3D reconstruction of SCPN injection volumes

To quantify injection volumes, cortical areas with AAV-labeled signal were manually outlined in FIJI/ImageJ, and the coordinates were transferred into CCFv3 reference atlas using the same custom MATLAB script described above. CCFv3-adapted cell coordinates were used to compute surface boundaries via Delaunay triangulation, followed by Laplacian smoothing to reconstruct and display the 3D injection volumes within the CCFv3 framework. The volumes were converted into a 100 μm voxel grid, retaining only enclosed voxels within the surface boundary, and injection volume was estimated by summing the volume of these enclosed voxels. To assess overlap with other volumes or selected ABA structures, enclosed voxels were compared to those in the target structure, and non-overlapping voxels were excluded. Percent overlap was calculated by dividing the number of overlapping voxels (overlap size) by the total number of voxels in the target structure.

### Quantification of axons within brainstem nuclei using StARQ

Utilizing the StARQ deep learning-based brainstem segmentation framework^33^, we fine-tuned the model on coronal brainstem sections with labeled SCPN axons (AAV-mediated anterograde labeling of SCPN in Cartpt-Cre and Npy-Cre mice) to automatically register and segment the brainstem regions of interest. Images were preprocessed using affine and non-rigid alignment. The fNissl signal was used for segmentation (registration channel) of the brainstem images to generate binary masks of brain regions with high confidence scores. Each predicted brainstem region (*Ri*) was then assigned a unique RGB color code for signal quantification, i.e., labeled SCPN axons. The binary mask for each segmented brainstem region (*Ri*) was used to filter out pixels in the corresponding signal channel of AAV+ SCPN axons (*Si*). The signal distribution (*Qi*) was computed by multiplying the binary mask (*Ri*) by the pixel intensities in the signal channel (*Si*) within the same section, effectively summing the pixel intensities within the masked region: *Qi* = (*Ri*·*Si*). The measured signals were normalized within each animal by dividing the signal for each region by the sum of signal across all regions in that animal. This in-animal normalization allowed for the comparison of relative signal distributions across different regions while accounting for variations in total signal strength between animals. Additionally, we measured the size of each region by calculating the total number of active pixels in the binary mask, ensuring that the area size was accounted for during signal analysis with no overt differences across experimental groups.

### Intersectional DREADD expression in SCPN subpopulations

For the conditional silencing of SCPN subpopulations using inhibitory DREADDs, Npy-Cre mice received bilateral injections of rAAV-DIO-FlpO (1.6 × 10^13^ GC/mL; Addgene, 87306-AAVrg^106^) either into the cerebral peduncles at P0 for M1.SCPN^off^ and Npy.CBN^off^ groups or into the cervical spinal cord at P2 for the Npy.CSN^Ctrl^ group. The Npy.CBN^off^ and Npy.CSN^Ctrl^ groups received a bilateral injection of AAV-fDIO-hM4Di-mCherry (1.41 × 10^12^ GC/mL; Vector Builder) at P1 into lateral cortex while the M1.SCPN^off^ group received a bilateral co-injection of AAV-fDIO-hM4Di-mCherry and AAV-hSyn-Cre (2.1 × 10^13^ GC/mL; Addgene, 105553-AAV1) at P1 into the M1 region of cortex. The AAV pEF1a-DIO-FLPo-WPRE-hGHpA was a gift from Li Zhang (Addgene viral prep # 87306-AAVrg; http://n2t.net/addgene:87306; RRID:Addgene_87306). The pENN.AAV.hSyn.Cre.WPRE.hGH was a gift from James M. Wilson (Addgene viral prep # 105553-AAV1; http://n2t.net/addgene:105553; RRID:Addgene_105553).

### Single pellet grasping

Adult Npy-Cre mice, which received intersectional viral labeling as pups, were handled for 5 minutes daily 5 days per week using a clear acrylic tube (⌀ 4.5 cm), which was left in the home cage over night to allow mice to familiarize themselves with the tube^107^. After a week of handling, baseline weights were recorded, and mice were placed on food restriction. Rationing started at 0.1g of food per gram of baseline body weight, which was adjusted over time to allow the mice to maintain weight above 85% of their baseline. Pellets were also added to each cage in addition to their daily rations to get mice familiar with them. Training for the task was divided into three phases: During habituation phase (lasting 1–3 days), mice were placed in an acrylic grasping chamber with a front-facing slit (based on Chen et al.^108^, gift of the Hollis lab) and allowed to explore for 10 minutes. Sugar pellets (20mg, chocolate flavor, Bio-Serv) were placed in front of the slit, and forceps were used to draw the mouse’s attention to the slit. Upon eating pellets regularly from the slit, they were fed pellets directly from the forceps. Once mice consistently ate from the forceps, they were determined to be ready for shaping. In the shaping phase (lasting 1–5 days), pellets were held with forceps in front of the mouse and were offered only after the mouse reached out and touched it with a paw. Once the mice were consistently touching the pellets and hand preference was established, pellets were placed on a pole (TR1.5 - Ø1/2” Optical Post, L = 1.5”, Thorlabs) positioned 1cm above the box floor and approximately 1.7cm from the slit, with a slight offset (0.5cm) to the side opposite from that of hand preference. This variant of the single pellet reaching task was specifically chosen to engage shoulder muscles during the reaching and grasping movements. Mice were rewarded for reaching towards the pole until they could consistently reach and touch the pellets, after which assistance was provided only when mice exhibited high levels of stress. Once mice were successfully reaching for pellets in an engaged manner and exhibited at least one successful grasp, we continued with the training phase (3–14 days). Reaching attempts were recorded as follows: Any touch of the pellet was recorded as an attempt. Additionally, successful reaches were counted when the pellet was successfully grasped and eaten. Sessions concluded after 25 attempts. Mice were considered fully trained after successfully grasping at least 30% of attempts on three consecutive days. If a mouse failed to meet this criterion in 10 sessions, it was classified as a non-learner and excluded from the experiment. After successful training, grasping performance was recorded for three consecutive days: To record baseline performance on day 1, mice received an intraperitoneal (i.p.) injection of 10 μL saline per 1 g body weight 30 min before recording. On day 2, mice received an i.p. injection of 10 μL DREADD ligand JHU37160 (J60; Hello Bio) for a final concentration of 1mg/kg body weight, and grasping performance was recorded 30 min after. Mice were again injected with saline 24h later, allowing sufficient time for the drug to be metabolized to record grasping performance after wash-out.

### Bioinformatics

Single-cell RNA-seq data were processed as recommended^109^. In brief, raw read files were processed and aligned to the mouse reference genome, mm10 (GENCODE vM23/Ensembl 98) using 10x Genomics Cell Ranger 6.0.1 count and aggr^110^. Using established R packages and custom-written code, cells with low read counts (empty droplets) or cells with a high number of mitochondrial gene products (±3x than median absolute deviation) were removed. Size-factor normalized logcounts were obtained^111,112^ and batch-corrected^113^. After integration of the three sample types (P1 SCPN, P3 SCPN, P3 CSN) using the top 2000 most variable genes with min. mean normalized expression of 0.001^114^, dimensionality reduction (UMAP) was done on the batch-corrected log counts.

For cluster stability analysis, gene expression counts from P1Ped and P3Ped samples were normalized using the SCTransform function in Seurat^115–117^, regressing out mitochondrial gene percentages. FastMNN^113^ was applied to remove batch effects, and the top 48 dimensions of the corrected embedding were used to construct a kNN graph with varying values of k (4, 6, 8, 10), which were refined into SNN graphs. Clusterings were computed on each SNN graph using the Louvain algorithm at resolutions of 0.2, 0.3, 0.4, and 0.5. To determine the most stable clustering, 80% of the dataset was subsampled 20 times, repeating the clustering process. Jaccard indices (JI) were calculated for each subsample, and stable clusters were defined using the AssignStableCluster from scclusteval R package^118^ as having a JI > 0.8 in at least 16 out of 20 subsamples. A k value of 8 and resolution of 0.4 produced the most stable clusters (n=6). Anchor cells between the P3SCc samples and the P1Ped and P3Ped samples were identified using the FindTransferAnchors function from Seurat. Using the identified anchors, the TransferData function was employed to classify P3SCc cells based on the stable cluster labels from the P1Ped and P3Ped dataset. The dataset was annotated with singleR^119,120^ using previously published single-cell data of the P0 mouse cortex to subset our dataset to Layer V neurons^23^. Axon extension specificity was defined as follows: clusters that were only present in the SCPN but not the CSN samples were annotated as “brainstem projecting”, while overlapping clusters were annotated as “spinal projecting”. Medial vs. lateral cortical location in our P1 sample was annotated using SingleR, leveraging microarray data from a previous study^20^ that compared medial vs. lateral SCPN at P1. This allowed us to label our P1 dataset based on transcriptional profiles matching the microarray time point. Marker genes between clusters were obtained using findMarker function in Seurat v4.0^116^.

Pseudotime trajectories were calculated from P1 to P3 (SCPN samples) using the destiny R package^121^. Diffusion components were calculated from the FastMNN-corrected embedding using DiffusionMap. This method captures cellular transitions through a non-linear dimensionality reduction technique^122^. Pseudotime was calculated for all cells using diffusion components with the DPT function, with the starting point set as the cell ranked first in the diffusion map’s eigenvector. To identify genes with continuous expression changes over pseudotime, a generalized additive model (GAM) was fitted to each gene’s expression using the gam function from the mgcv R package^123^. The data were normalized with SCTransform, and principal component analysis (PCA) was performed to reduce dimensionality, followed by mutual nearest neighbors (MNN) embedding and UMAP visualization. Diffusion maps were computed on the MNN coordinates to calculate pseudotime, which was then visualized using ggplot2.

Mapping to mouse adult single-cell data of whole cortex^24^ was done as follows: The gene expression matrix from the P1-P3 developmental dataset was combined with a mouse adult single-cell dataset (P56). FastMNN was used to align cells across the datasets, correcting for batch effects while preserving biological variation. The top 30 MNN-corrected dimensions were used to construct a kNN graph with the FindNeighbors function, which was refined into an SNN graph. Clusters were identified on this SNN graph using the Louvain algorithm in Seurat with a resolution of 0.8. For visualization, Uniform Manifold Approximation and Projection (UMAP) was computed on the MNN-corrected embedding using the RunUMAP function.

### Statistical analysis

Statistical analysis other than those related to the single-cell RNA-seq data was performed in Prism 10.3 (GraphPad Software) and R version 4.4.1. Ordinary one-way ANOVA with Tukey multiple comparisons was used for group comparisons (within-group differences in cortical locations, behavior, and across-group differences in brainstem regions of interest). For the comparison of retrogradely labeled SCPN vs. CSN at P4, mixed-effects models with restricted maximum likelihood estimation (REML) were applied to accommodate variability in bregma level alignment. A two-tailed Wilcoxon rank-sum test (Mann-Whitney U test) was used to compare expression levels of Npy/Cartpt at P1 vs. P3 in the scRNAseq dataset (only cells with expression > 0 were included). In all bar graphs and box plots, dots represent individual animals. The threshold for significance for all experiments was set at *p < 0.05. Smaller p values were represented as **p < 0.01 and ***p < 0.001. In bar graphs, all data are plotted as mean ± SEM. In box plot graphs, data are represented as median ± 25th percentile (box) and min/max (whiskers).

## Extended Data

## Supplementary Material

1

## Data Availability

All raw sequencing data reported in this paper have been deposited at GEO and are publicly available; NCBI GEO accession number: GSE270265. Custom FIJI/MATLAB pipelines for 3D reconstruction of injection volumes and cell coordinates within CCFv3 space available on https://github.com/jkaiser87/ (VOL3D for injection volume, CELL3D for cell coordinates).
