# Supplementary material for: Developmental molecular signatures define de novo cortico-brainstem circuit for skilled forelimb movement
Source: Res Sq. 2025 Mar 26:rs.3.rs-6150344. Preprint. [Version 1] doi: 10.21203/rs.3.rs-6150344/v1 (PMC11975033; doi:10.21203/rs.3.rs-6150344/v1)
Supplement: 1 [file NIHPPRS6150344V1-supplement-1.pdf]

Supplementary Video 1 (Optically cleared brainstem related to Fig. 5)

Supplementary Table 1 – Top differentially expressed genes (attached xls file)

Supplementary Table 2 – Quantification of axonal distribution across brainstem nuclei (attached xls file)

Supplementary Table 3 – Brainstem Region Abbreviations

High.level	Region	Relation	safe_name
Midbrain	DR	behavior	Dorsal nucleus raphe
Midbrain	IPN	behavior	Interpeduncular nucleus
Midbrain	AT	motor	Anterior tegmental nucleus
Midbrain	III	motor	Oculomotor nucleus
Midbrain	MRN	motor	Midbrain reticular nucleus
Midbrain	ND	motor	Nucleus of Darkschewitsch
Midbrain	PAG	motor	Periaqueductal gray
Midbrain	RN	motor	Red nucleus
Midbrain	SC	motor	Superior colliculus motor related
Midbrain	SN	motor	Substantia nigra reticular part
Midbrain	VTA	motor	Ventral tegmental area
Midbrain	IC	sensory	Inferior colliculus
Midbrain	MEV	sensory	Midbrain trigeminal nucleus
Midbrain	PBG	sensory	Parabigeminal nucleus
Pons	CS	behavior	Superior central nucleus raphe
Pons	LDT	behavior	Laterodorsal tegmental nucleus
Pons	PRN	behavior	Pontine reticular nucleus
Pons	LC	behavior	Locus ceruleus
Pons	DTN	motor	Dorsal tegmental nucleus
Pons	PCG	motor	Pontine central gray
Pons	PG	motor	Pontine gray
Pons	TRN	motor	Tegmental reticular nucleus
Pons	V	motor	Motor nucleus of trigeminal
Pons	NLL	sensory	Nucleus of the lateral lemniscus
Pons	PB	sensory	Parabrachial nucleus
Pons	PSV	sensory	Principal sensory nucleus of the trigeminal
Pons	SOC	sensory	Superior olivary complex
Medulla	RM	behavior	Nucleus raphe magnus
Medulla	RO	behavior	Nucleus raphe obscurus
Medulla	AMB	motor	Nucleus ambiguus
Medulla	DMX	motor	Dorsal motor nucleus of the vagus nerve
Medulla	GRN	motor	Gigantocellular reticular nucleus
Medulla	IO	motor	Inferior olivary complex
Medulla	IRN	motor	Intermediate reticular nucleus
Medulla	LAV	motor	Lateral vestibular nucleus
Medulla	LIN	motor	Linear nucleus of the medulla
Medulla	LRNm	motor	Lateral reticular nucleus magnocellular part

Medulla	MARN	motor	Magnocellular reticular nucleus
Medulla	MDRNd	motor	Medullary reticular nucleus dorsal part
Medulla	MDRNv	motor	Medullary reticular nucleus ventral part
Medulla	MV	motor	Medial vestibular nucleus
Medulla	PARN	motor	Parvicellular reticular nucleus
Medulla	PGRNd	motor	Paragigantocellular reticular nucleus dorsal part
Medulla	PGRNl	motor	Paragigantocellular reticular nucleus lateral part
Medulla	PPY	motor	Parapyramidal nucleus
Medulla	PRP	motor	Nucleus prepositus
Medulla	SPIV	motor	Spinal vestibular nucleus
Medulla	SUV	motor	Superior vestibular nucleus
Medulla	VII	motor	Facial motor nucleus
Medulla	VI_AN	motor	Abducens nucleus
Medulla	X	motor	Nucleus x
Medulla	XII	motor	Hypoglossal nucleus
Medulla	VNC	motor	Vestibular nuclei
Medulla	CN	sensory	Cochlear nuclei
Medulla	CU	sensory	Cuneate nucleus
Medulla	ECU	sensory	External cuneate nucleus
Medulla	NTB	sensory	Nucleus of the trapezoid body
Medulla	NTS	sensory	Nucleus of the solitary tract
Medulla	SPV	sensory	Spinal nucleus of the trigeminal caudal part
Medulla	AP	sensory	Area postrema
Medulla	GR	sensory	Gracile nucleus

Supplementary Table 4 – Allen Brain Atlas Isocortex structure Abbreviations

Abbreviation	Full Name
MOs	Secondary Motor Area
MOp	Primary Motor Area
SSp	Primary Somatosensory Area
SSs	Supplemental Somatosensory Area
AUD	Auditory Areas
VIS	Visual Areas
AI	Agranular Insular Area
ACA	Anterior Cingulate Area
